# How sphingolipids affect T cells in the resolution of inflammation

**DOI:** 10.3389/fphar.2022.1002915

**Published:** 2022-09-13

**Authors:** Jennifer Christina Hartel, Nadine Merz, Sabine Grösch

**Affiliations:** ^1^ Institute of Clinical Pharmacology, Goethe-University Frankfurt. Frankfurt am Main, Frankfurt, Germany; ^2^ Department of Life Sciences, Goethe-University Frankfurt, Frankfurt, Germany; ^3^ Fraunhofer Institute for Translational Medicine and Pharmacology ITMP, Frankfurt, Germany

**Keywords:** ceramides, gangliosides, S1P, lipid rafts, treg, Th17, bioactive lipids

## Abstract

The concept of proper resolution of inflammation rather than counteracting it, gained a lot of attention in the past few years. Re-assembly of tissue and cell homeostasis as well as establishment of adaptive immunity after inflammatory processes are the key events of resolution. Neutrophiles and macrophages are well described as promotors of resolution, but the role of T cells is poorly reviewed. It is also broadly known that sphingolipids and their imbalance influence membrane fluidity and cell signalling pathways resulting in inflammation associated diseases like inflammatory bowel disease (IBD), atherosclerosis or diabetes. In this review we highlight the role of sphingolipids in T cells in the context of resolution of inflammation to create an insight into new possible therapeutical approaches.

## The innate and adaptive immune system

**FIGURE 1:**
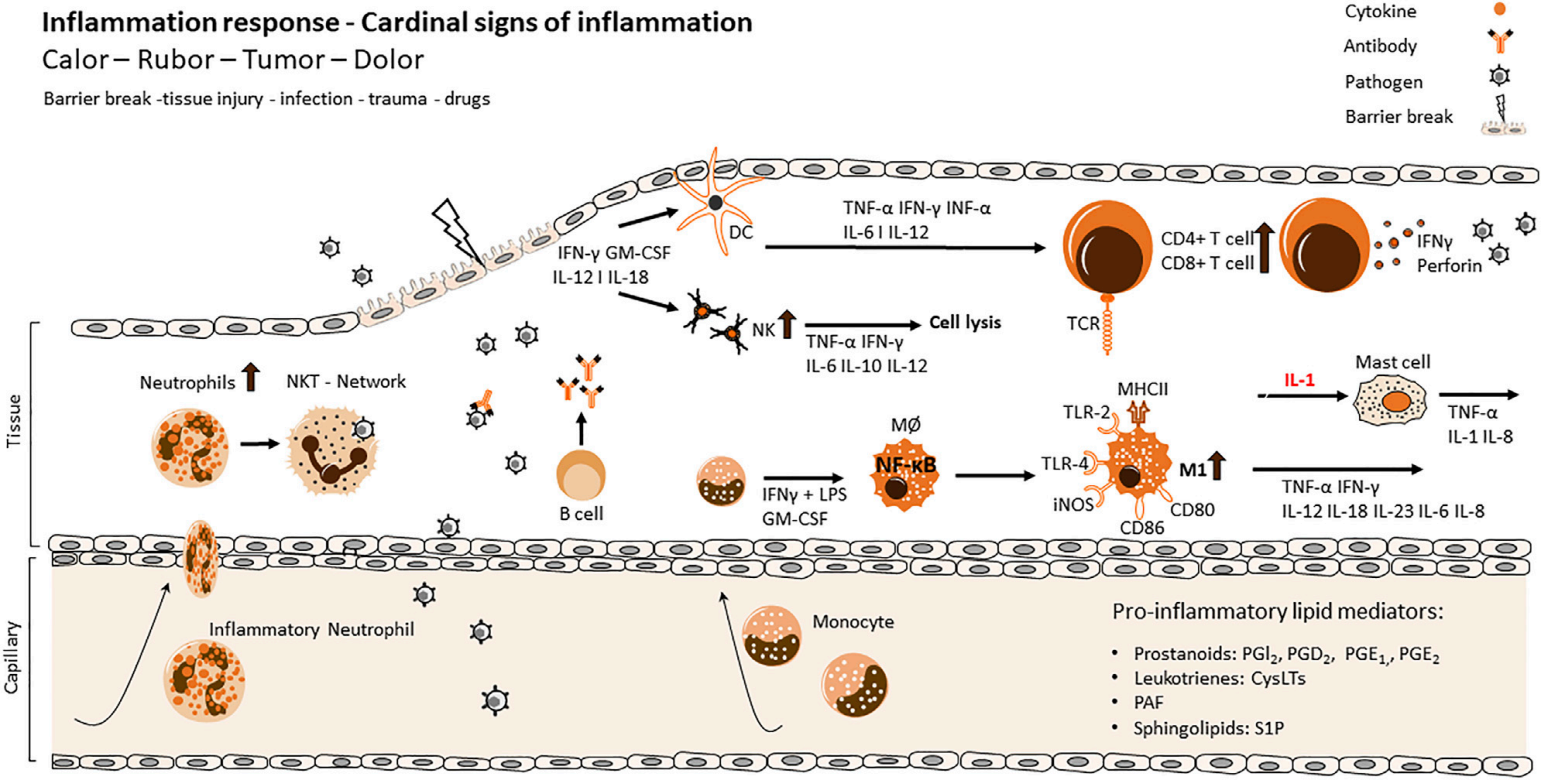
Cellular and molecular processes of inflammation and resolution via innate and adaptive immune response. Main actors of the different stages of the inflammatory process. Tissue injury of the primary immunological barrier is followed by the invasion of pathogens and viruses. Infiltration initiates an activation of highly coordinated receptors like TLRs and CD36 at the surface of innate immune cells and triggers the recruitment of further immune cells of the innate or adaptive immune system. While the inflammatory response evolves, several cellular functions are exerted contemporaneously like the release of toxic substances, phagocytosis, the release of pro-inflammatory cytokines and the initiation of antigen recognition *via* APC cells. Maturated dermal DCs migrate into lymph nodes in order to prime naïve T cells which results in an influx and upregulation of CD4+/CD8+ cells and production of IFNγ and perforin as an adaptive immune response. The maturation of DCs results in an upregulation of MHC class II expressing molecules, CD40, CD80, CD86 as well as the production of cytokines like IL12, which activates NK cells to produce IFN-γ and induces the differentiation of Th1 cells. The production of IFNγ and IL2 enhance DC maturation, simultaneously facilitating the clonal expansion of antigen-specific naive T cells, ultimately amplifying the adaptive immune response. IL, interleukin; IFN, interferon; TNF, tumor necrosis factor; TGF, transforming growth factor; Th, helper T cell; Treg, regulatory T cell; TCR, T cell receptor; NF-κB, nuclear factor kappa-light-chain-enhancer of activated B cell; TLR, toll-like receptor.

The immune system is a large network consisting of an innate and an adaptive part. Both systems work together and intertwine with each other to defend the body from germs or foreign substances (Figure 1) (Yatim and Lakkis, 2015; Hillion et al., 2020). The skin as part of the innate immune system is the first defence against pathogens and protects our body from foreign entries. When pathogens passed this barrier the innate immune system then activates a cascade of signalling pathways and immune cells from both, the innate and the adaptive, immune systems to fight off the danger (Smith et al., 2019).

Pathogens can be neutralized or directly killed by cells of the innate immune system (Macrophages, Natural killer cells etc.), but the scope of function of the innate immune cells goes beyond. Bacterial cell wall components or viral particles are processed by cells of the innate system and presented as antigens to the adaptive immune system. Furthermore, the innate immune cells recruit adaptive immune cells from the blood to the site of inflammation by secretion of chemokines and cytokines.

The adaptive immune system consists of T lymphocytes, B lymphocytes and the antibodies that are produced by the B cells (Buchholz et al., 2016; Bradley and Thomas, 2019; Wang et al., 2020). B cells come from and mature in the bone marrow. They are activated by a subpopulation of T cells (T helper cells) via antigens that bind at their surface immunoglobulin (Ig). Upon activation B cells then release soluble antibodies. The antibody release enables the humoral immune response consisting of pathogen neutralisation, opsonization and complement activation. The complement system enzymatically facilitates lysis and phagocytosis of pathogens (Merle et al., 2015). Antibodies also prevent bacterial adherence including binding to the pathogen (Forthal, 2014).

T cells derive from the bone marrow and are then moved through the blood for maturation in the thymus. They have a broad spectrum of functions that are determined by their surface expression pattern that divides these cells into different subpopulations (Romagnani, 2014). The activation and maturation of those cells is mediated via the T Cell Receptor (TCR). They have to undergo a checkpoint to ensure only non-self-antigen responsive cells are selected (Fife and Bluestone, 2008). Negative selection of T cells removes self-reactive cells from the body (Palmer, 2003), positive selection assures that only T cells with a TCR that can recognize foreign antigens via MHC stimulation will mature (Klein et al., 2014). This process takes places in the thymus and occurs with cells that already express the TCR and both co-receptors Cluster of Differentiation (CD) 4 and 8.

## Inflammation processes and proper resolution

Under normal circumstances, the process of inflammation is a mechanism to defend the body from foreign danger like pathogens, toxins or damaged cells. Usually this process is managed within days and finished by resolution of this acute inflammation (Varela et al., 2018). Inflammatory cells, such as macrophages, neutrophils, B cells and T cells, are recruited and send to the site of pathogen infiltration to act against the those pathogens to heal the injury and thereby restoring tissue homeostasis (Figure 1) (Schnoor et al., 2016).

Local, acute inflammation in the tissue is characterized by Galen’s prominent five properties: rubor (redness), tumor (swelling), dolor (pain), calor (heat) and function laesa (impaired function). These characteristics are triggered by the immune cell response to the infectious or sterile damage. Receptors on the surface of immune cells can recognize harmful stimuli via different receptors. Infectious stimuli are so called PAMPs (pathogen-associated molecular patterns) that activate a variety of PRRs (pattern-recognition receptors) in immune and non-immune cells, but additionally endogenous signals also known as DAMPs (danger-associated molecular patterns) can be responsible for inflammatory responses (Walsh et al., 2013; Eppensteiner et al., 2019; Zindel and Kubes, 2020; Murao et al., 2021). Toll-like receptors (TLRs) are one class of these PRR. When they get activated, different inflammatory signalling cascades in immune or non-immune cells are triggered, resulting in the translocation of the nuclear factor 'kappa-light-chain-enhancer' of activated B-cells (NF-κB), activator protein 1 (AP-1) or interferon regulatory factor 3 (IRF3) from cytosol to the nucleus, thereby leading to a transcriptional regulation of the immune response (Kawai and Akira, 2007; Liu W. et al., 2009). The activation of these pathways leads to the release of pro-inflammatory cytokines like Interferon-Gamma (IFN-γ), Interleukin 1β (IL1β) or IL6 and pro-inflammatory lipid mediators like Platelet-activating factor (PAF, 1-O-alkyl-2-acetyl-sn-glycero3-phosphocholine), leukotriens, prostanoids or sphingolipids (Lawrence, 2009; Schauberger et al., 2016; Srivastava and Baig, 2018; Yanai et al., 2018). Interactions of cytokines with their receptors on immune cells activate signalling pathways including NF-κB, mitogen-activated protein kinase (MAPK), januskinase- signal transducers and activators of transcription (JAK-STAT) that promote further cytokine production, proliferation and differentiation of immune cell to specific subtypes (Moens et al., 2013; Fang et al., 2018; Salas et al., 2020). PAF is produced by various cells including neutrophils, eosinophils, endothelial cells and fibroblasts and induces platelet aggregation and leukocyte degranulation and adhesion (Pałgan and Bartuzi, 2015). Leukotrienes (Cysteinyl leukotrienes (CystLTs), leukotriene B4 (LTB4), leukotriene C4 (LTC4)) are produced by leukocytes such as macrophages, eosinophils, basophils and are important for leukocyte influx and vascular permeability (Rådmark et al., 2015; Schauberger et al., 2016). Prostanoids are produced by almost every cell type and are crucial for chemotaxis, activation of immune cells, act on platelet aggregation and vascular smooth muscle cells (Schauberger et al., 2016). S1P (sphingosine-1 phosphate) is the best known pro-inflammatory sphingolipid and involved in chemotaxis as well as activation of various immune cells (see also chapter 6.4) (Sukocheva et al., 2020).

**FIGURE 2:**
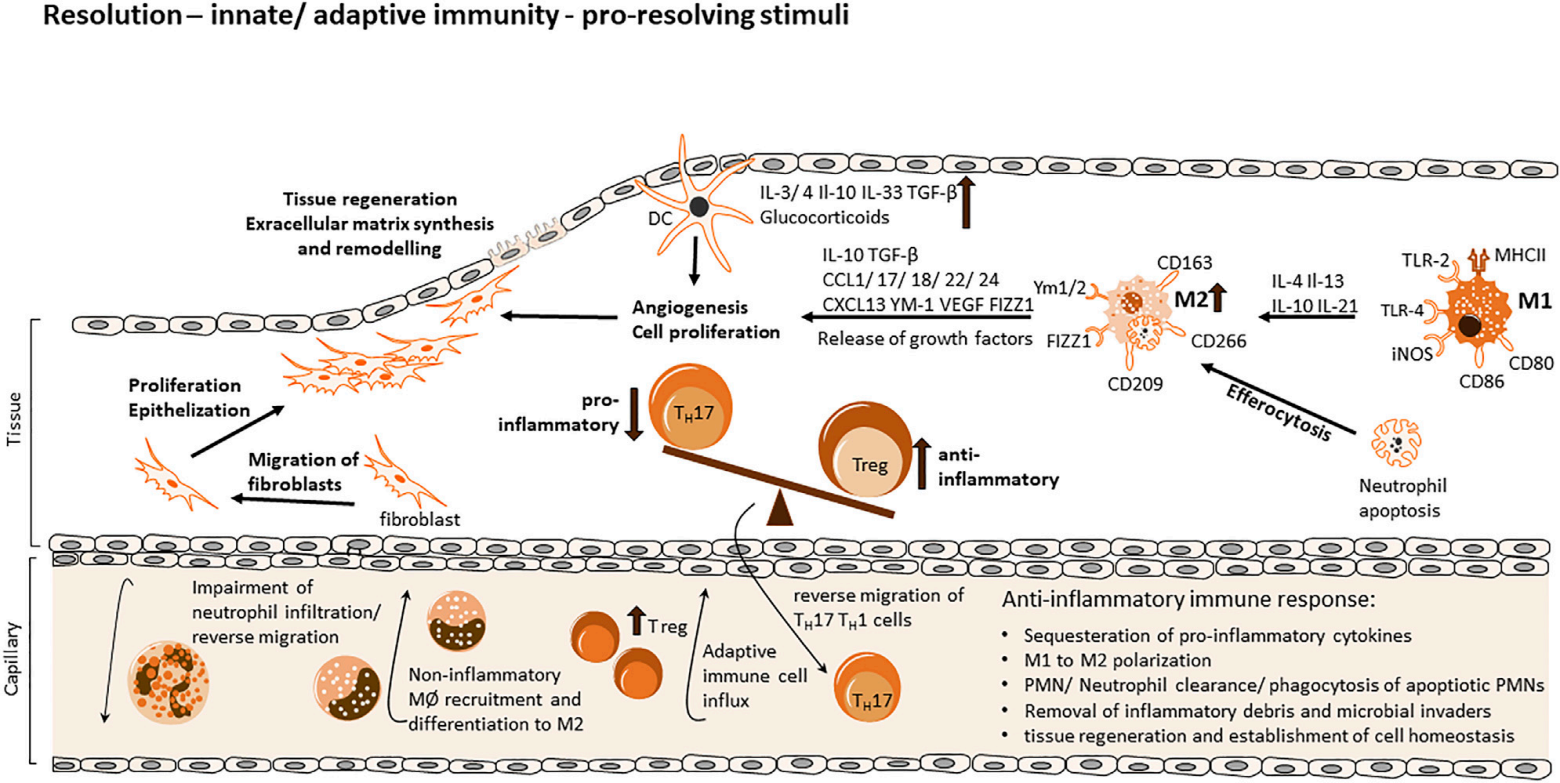
Cellular and molecular processes of inflammation and resolution via innate and adaptive immune response. During the evolution of the inflammatory response, various mechanisms facilitate a shift to a resolving cell-program phenotype via generation of anti-inflammatory and therefore pro-resolving mediators, creating a favorable environment for the resolution phase and return to tissue homeostasis and regeneration. Diverse mechanisms consist of the sequestration of pro-inflammatory cytokines, proteolysis of chemokines and degradation via NETs (neutrophil extracellular traps) thereby re-modelling chemokine gradients and impairing the influx of neutrophils. Apoptotic neutrophils release pro-resolving mediators, leading to an inhibition of continued neutrophil infiltration and a non-inflammatory monocyte recruitment upon differentiation to M2 macrophages. Neutrophils further promote their dismissal *via find me* and *eat me* signals, which attract scavengers and allow identification of the apoptotic cell. Anti-inflammatory mediators facilitate the polarization of M1 macrophages towards a resolution-phase phenotype, promoting efferocytosis and the expression/release of growth factors for tissue regeneration, enable the migration of fibroblasts, extracellular matrix synthesis and the remodeling of tissue to a final re-establishment of cell homeostasis. The influx of Tregs from the adaptive immune response re-program inflammatory responses and mediators and upregulation of anti-inflammatory Tregs in contrast to Th17 cells as well as their reverse migration promote resolution further.

To restore the pre-inflammation status of the tissue, immune cells must be removed from the inflammatory site, inflammatory signalling processes have to be resolved and chemokine gradients have to be diluted to prevent further migration of immune cells (Figure 2). Chemokines at the site of inflammation are cleaved by proteolysis and sequestration (Ye, 2013; Chongsathidkiet et al., 2018; Tao et al., 2019; Sallenave and Guillot, 2020). Neutrophils are the first cells to be egressed from the tissue (Greenlee-Wacker, 2016). They undergo Fas-ligand induced apoptosis via tumor necrosis factor (TNF) receptor interactions that lead to phosphoinositide 3-kinase (PI3K)-activation, they produce reactive oxygen species (ROS) (Salamone et al., 2001; Croker et al., 2011). Macrophages are responsible for the release of Fas-ligand and TNF and the clearance of neutrophils that went apoptotic (Brown and Savill, 1999). During this efferocytosis the pro-inflammatory expression pattern (M1) of macrophages change to an anti-inflammatory pattern (M2) (Kohno et al., 2021). M2 macrophages are characterized by their ability to release immunosuppressive cytokines like IL10, transforming growth factor β (TGFβ) and vascular endothelial growth factor (VEGF) (Orecchioni et al., 2019).

Anti-inflammatory signals are beneficial for tissue homeostasis. Stromal cells, macrophages and progenitor or stem cells must work together in order to impede scar formation or fibrosis (Figure 2). TGFβ released from macrophages promote fibroblast differentiation into myofibroblasts through regulation of ECM remodelling (Caja et al., 2018). VEGF signalling leads to angiogenesis and recovery of normal oxygen supply (Shibuya, 2011). Also mesenchymal stem cells (MSC) affect inflammation by the production of growth factors and chemokines that influence macrophage polarization, maturation and migration (Zhao et al., 2020). MSCs promote phagocytosis of macrophages and their differentiation by effecting the metabolism of the macrophages via a prostaglandin E2 (PGE2) dependant mechanism (Vasandan et al., 2016). Polarization of M1 type macrophages to a M2 type can also be enhanced by MSCs (Liao et al., 2020).

Dysfunction or -regulation of these processes can shift acute inflammation to a chronic status and are the cause of many diseases like inflammatory bowel disease (IBD), chronic airway inflammation, stromal keratitis, rheumatoid arthritis and even cancer.

## T cell subsets

T cells have a broad spectrum of functions. CD8 expressing T cells are also called cytotoxic T cells because of their capability of killing pathogens and cancer cells. Cytotoxic T cells have to be stimulated by antigen-presenting cells (APC) via MHC class I in order to mature (Kratky et al., 2011). This happens in the nature of an infection and causes these cells to proliferate and fight off intracellular dangers (Cox and Zajac, 2010). CD8 cells are capable of direct killing of infected cells via induction of apoptosis in those cells through the secretion of perforin and granzymes (Voskoboinik et al., 2015). Perforins form pores into the target’s cell membrane and enable the entrance of granzymes (Osińska et al., 2014). Granzymes are a group of serine proteases that cleave viral and cellular proteins of the infected target cells thereby killing it (Trapani, 2001). Apoptotic target cells are then phagocytized by macrophages (Kourtzelis et al., 2020). Another possibility of target cell killing is the induction of apoptosis via Fas-FasL interactions (Yamada et al., 2017). Upon Fas activation a signalling cascade is activated and caspase proteases are induced following apoptosis of these cells (Aouad et al., 2004; Sobrido-Cameán and Barreiro-Iglesias, 2018).

CD4 cells are often characterized as T helper cells (Th). Those cells regulate immune and non-immune cells via the production and release of cytokines and recognize antigens via MHC class II molecules. There is a variety of different T helper cell subsets, that is, described by their cytokine release and transcriptional profile. On the one hand, Th1 cells are prominent to produce IFNγ, TNFβ, TNFα and IL2 and they also express the transcription factor T-bet (T-box expressed in T cells) (Viallard et al., 1999; Szabo et al., 2000; Hwang et al., 2005; Solomou et al., 2006; Kisuya et al., 2019; Artham et al., 2020). On the other hand, TH2 cells do not release IFNγ, but IL4, IL5 and IL13 and they produce the transcription factors GATA binding protein 3 (GATA-3) and c-Maf (musculoaponeurotic fibrosarcoma) (McKenzie et al., 1998; Kanhere et al., 2012; Paul, 2015; Imbratta et al., 2020). Both subpopulations express different chemokine receptors, Th1 cells express C-C chemokine receptor type 5 (CCR5) and CXCR3, TH2 cells express CCR4 and CCR8 (Zingoni et al., 1998; Turner et al., 2007; Yoshie and Matsushima, 2015; Watanabe et al., 2020). Th1 cells are mainly responsible for the recruitment and activation of macrophages by IFNγ release and TH2 cells activate B cells via IL4 and IL13, but also eosinophils via IL5.

There is one additional major subpopulation among the CD4 effector cells, that is, called Th17 cells. Those cells mainly release IL17A to activate macrophages, endothelial and epithelial cells and fibroblasts, as well as CXCL8 to stimulate neutrophils and exhibit pro-inflammatory functions (Pelletier et al., 2010; Li J. et al., 2013; Brembilla et al., 2013; Tabarkiewicz et al., 2015; Zhang et al., 2021; Muench et al., 2022). RAR-related orphan receptor gamma (ROR(γ)t) is a transcription factor, that is, solely expressed by Th17 cells (Steinmetz et al., 2011).

Besides their effector functions in inflammatory processes, there is one specialized CD4 subset, that has a more regulatory function. Regulatory T cells (Tregs) are a subpopulation of CD4 expressing cells that additionally express high amounts of CD25, the alpha-subunit of the IL2 receptor (Kmieciak et al., 2009). They are also often categorized by their expression of the transcription factor forkhead box P3 (FOXP3) which is a prominent Treg marker (Rudensky, 2011). It is important for both, regulatory and developmental pathways in Tregs. Tregs are known for their immune-suppressive function and behaviour (Wan, 2010). The production of the anti-inflammatory cytokine IL10 is one of the main tasks of Tregs and a key player in suppressive function (Ng et al., 2013). The process of self-tolerance is tightly regulated by Tregs and disturbances in this processes lead to autoimmune diseases (Sakaguchi et al., 2008; Yang et al., 2015). Because of their immunosuppressive function, Tregs are a key mediator in resolution of inflammation. They promote macrophage efferocytosis and modulate monocyte macrophage differentiation thereby driving the resolution of inflammation (Weirather et al., 2014; Proto et al., 2018).

## T cell activation

The TCR is a heterodimer complex that has two TCR chains and six CD3 chains also connected with different components like Co-receptors, kinases and ligands. Most T cells express the α- and β- TCR chain and are referred to as normal T cells, whereas T cells that express γ- and δ- TCR chain receptors are called γδ- T cells (Nanno et al., 2007). They only make up 0.5–5% of the T lymphocytes (Carding and Egan, 2002). The variable domains of the α and β, γ δ respectively, are responsible for the specificity of the TCR and distinguish the different antigens presented by the Major Histocompatibility complex (MHC). TCRα/TCRβ and TCRγ/TCRδ heterodimers form complexes with the CD3γ/ε and/or CD3δ/ε heterodimer molecules, as well as CD3ζ homodimers (Birnbaum et al., 2014).

T cells can be generally classified via their cell surface molecules CD4 and 8, respectively. Both markers are co-stimulatory proteins of the TCR. They are important mediators of the MHC peptide recognition cascade and they are also important for the maturation process of T cells in the thymus (Tikhonova et al., 2012). They are single molecules with an extracellular and a transmembrane region and an intracellular tail (Leahy, 1995). The intracellular tail enables interaction with the lymphocyte-specific protein tyrosine kinase (LCK) which initiates the signalling cascade upon activation of the TCR (Artyomov et al., 2010). CD4 can recognize MHC class I molecules, whereas CD8 cells bind to MHC class II molecules (Li Y. et al., 2013).

The activation of TCR is only possible when not only CD3 or CD4/8 are stimulated but also CD28 (Linsley and Ledbetter, 1993). It is a cell surface monomeric protein that binds to CD80 and CD86 of APCs in order to activate phosphoinositide-3-kinases (PI3K) (Garçon et al., 2008). PI3K catalyzes the conversion of phosphatdiylinositol-4,5-bisphosphate (PIP2) to phosphatidylinositol-3,4,5-trisphosphate (PIP3) which then activates phospholipase C-γ (PLC-γ) in the TCR activation machinery (Falasca et al., 1998). CD28 also enhances the production of IL4 and IL10 via TCR activation and CD40L binding (Hünig et al., 2010).

Upon stimulation of the TCR CD4/8 mediated LCK activation leads to the cascade of ZAP-70 phosphorylation and subsequent activation of the linker activator of T cells (LAT) (Lo et al., 2018). LAT as well as CD28, activate PI3K to form PIP2, but LAT can also activate PLC-γ directly (Balagopalan et al., 2015). Signalling pathways of TCR lead to NFκB, AP-1 and NFAT activation, which then promotes cell proliferation, differentiation and cell survival (Smith-Garvin et al., 2009).

The organization of the TCR’s activation takes place in so-called lipid rafts, often also called micro-domains or detergent-resistant membranes (DRM) (Zumerle et al., 2017). Lipid rafts are specialized compartments in the cell membrane with a high density of sphingomyelins, cholesterols and glycosphingolipids (Lingwood and Simons, 2010; Simons and Sampaio, 2011; Regen, 2020). They are cellular signalling hubs, bringing receptors closely together, kinases and second messengers thereby transforming a signal from the membrane to intracellular signalling cascades. The assembly of TCR/CD3, CD28, PKCθ and LCK is defined as supramolecular activation cluster (SMAC) with a central region (c-SMAC) and a peripheral region (p-SMAC) (Zumerle et al., 2017).

## Insights into sphingolipid metabolism and function

**FIGURE 3:**
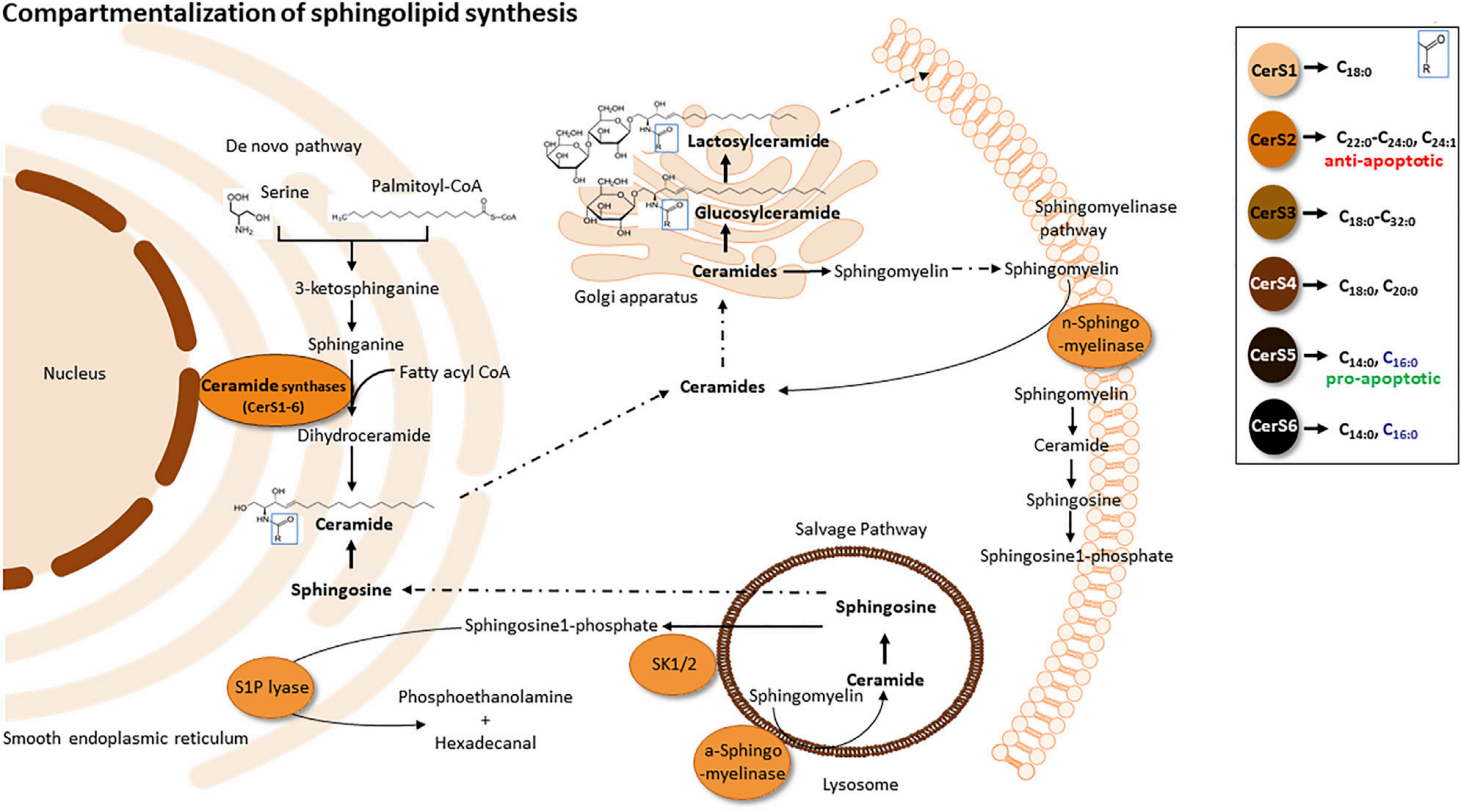
Compartmentalization of sphingolipid synthesis. *De novo* sphingolipid synthesis (anabolic pathway) is located at the smooth ER and ER-associated membranes - perinuclear membrane and mitochondria associated membranes (MAMs). Sphingolipid synthesis starts via the condensation of L-serine and palmitoyl co-enzyme A (CoA) to form 3-ketosphinganine by serine palmitoyltransferase. Subsequently, 3-ketosphinganine reductase reduces 3-ketosphinganine to sphinganine, which is acylated in order to from dihydroceramide by ceramide synthase. Dihydroceramide is afterwards oxidized by a desaturase, which results in ceramide formation. Ceramide is transported from the ER to the Golgi apparatus and is converted into sphingomyelin by sphingomyelin synthase or glycosphingolipids by ceramide glucosyltransferase. Sphingomyelin and complex glycosphingolipids synthesized in the Golgi apparatus are transported to the plasma membrane. Within the plasma membrane and other cell compartments, sphingomyelin are hydrolyzed by acid/neutral sphingomyelinases (SMases) to yield ceramide. Ceramide can be hydrolyzed by ceramideases (Cdases) to form sphingosine, which can be phosphorylated by sphingosine kinase (SphK) to generate sphingosine-1-phosphate (S1P). S1P can then be cleaved by S1P lyase to fatty aldehyde and phosphoethanolamine. Alternatively, S1P can be dephosphorylated back to sphingosine by phosphatases.

As lipid rafts are characterized as structures enriched in sphingomyelins and glycosphingolipids, the role of sphingolipids (Sph) is very crucial (Bieberich, 2018). Sphs are one of the most important classes of membrane lipids. They owe their name to the mythological sphinx after they were first discovered in brain tissue in the 1870s (Thudichum, 1962). Sphs commonly consist of a sphingoid base, like sphingosine or sphinganine, as backbone, at which via an amid-bound a second fatty acyl chain of different chain length is added. Further, the C1-hydroxygroup could be connected to different charged groups like phosphate, glucose/galactose or phosphocholine, resulting in Ceramide-1-phosphate, Glucosyl-Ceramide (GluCer), Galactosyl-Ceramide (GalCer) or sphingomyelin (SM). GluCer can further be processed to Lactosyl-Ceramide (LacCer) and complex Gangliosides (GMs). A nice overview of the sphingolipid pathway is given in (Rajasagi and Rouse, 2018). The *de novo* synthesis of ceramides takes place in the Endoplasmatic Reticulum (ER) afterwards ceramides are transported to the Golgi apparatus where the synthesis of complex sphingolipids takes place (Figure 3). The first step of the sphingolipid *de novo* synthesis is mediated by the serine palmitoyltransferase (SPT) which condensates serine and palmitoyl-CoA to generate 3-ketosphinganine, that is, directly reduced to sphinganine by the 3-ketophinganine reductase. Six isoforms of ceramide synthases (CerS1-6) add a second acyl-chain of different chain length to sphinganine/sphingosine to generate dihydro-ceramides or ceramides, respectively. CerS1 and CerS4 add C18/C20 fatty acids, CerS2 C22-C26, CerS3 C18-C32 and CerS5 and CerS6 use mainly C14/C16 fatty acids as substrate (Figure 3). Ceramide synthases are regulated and expressed tissue specific and each ceramide has definite cellular effects (Brachtendorf et al., 2019 und Wegner et al., 2016). Sphingolipids could also be recycled in the salvage pathway, where sphingosine can be obtained from constitutive degradation of sphingomyelin or glycosphingolipids either at the plasma membrane or in the late endosomes and lysosomes by the action of sphingomyelinases, glycosidases and ceramidases. Sphingosine is then either N-acetylated by CerS to form ceramides, phosphorylated by sphingosine-kinases to sphingosine-1-phosphate (S1P), or degraded to phosphatidylethanolamine and fatty aldehyde by lyases. S1P regulates many cellular functions via binding to G protein-coupled receptors S1P1-5. Those receptors reside in different cell types and tissues and upon their stimulation trigger signalling cascades for proliferation, survival, migration and vaso-regulative pathways (Schwab and Cyster, 2007; Obinata and Hla, 2019). The impact of membrane located sphingolipids on cells depends on their basic structure. Ceramides, for example, can affect cellular pathways and diseases differentially through the length of their fatty acid chains (Grösch et al., 2012). On the one hand, the amphilic properties of short chain ceramides result in membrane permeability and enable them to translocate freely into the cytosol. On the other hand, long chain ceramides stay tightly bound to the membrane and cannot pass as freely. Very long chain ceramides are responsible for the epidermal barrier function and can be found in the skin (Li et al., 2007). Specific lipid and protein interactions in membranes are crucial for signal transduction and can be influenced also by the chain length, saturation of the acyl chain or their polar head groups (Quinn, 2014; Uche et al., 2021). Therefore, the biophysical properties of membrane lipids can facilitate or impede receptor signalling.

Sphs control and influence major cellular events such as apoptosis, cell growth and proliferation, signal transduction and differentiation (Cerbón et al., 2018; Xie et al., 2019; Hadas et al., 2020; Jakobi et al., 2020; Loberto et al., 2020; Mignard et al., 2020; Muthusamy et al., 2020; Monasterio et al., 2021; Capolupo et al., 2022). Therefore, a dysregulation of these processes have also an impact on chronic inflammation which we want to highlight in the next sections.

## The impact of sphingolipids on T cells and their role in resolution of inflammation

In this chapter we want to give an overview how T cells affect resolution of inflammation and how sphingolipids can influence these mechanisms. As mentioned above, T cells have different functions in inflammation and can either enhance or contribute to the resolution of inflammatory lesions. While T helper cells seem to increase and sustain chronic inflammation, Tregs display an important factor in the resolution process.

### Role of sphingolipids in treg development and function

Early study showed that the adoptive transfer of Tregs into immune-deficient mice with Th1 and Th2 induced colitis leads to resolution of inflammation and reappearance of normal intestinal architecture (Table 1) (Mottet et al., 2003). Nowadays, we know that Tregs promote macrophage efferocytosis by production and release of IL13 that activates IL10 production in macrophages (Proto et al., 2018). IL10 release enhances efferocytosis and immunosuppression but can also promote the M1 to M2 macrophage phenotype class switch. As a hallmark of Treg cells the transcriptional program is controlled by Foxp3 that reduces the expression of sphingomyelin synthase 1 (SMS1). This leads to an increase in ceramide level, among others C18-ceramide (Apostolidis et al., 2016). C18-ceramide interferes strongly with the SET protein (originally named inhibitor 2 of protein phosphatase 2 A) which reduces the interaction between SET and PP2A and constraints its inhibitory action on the PP2A complex (Mukhopadhyay et al., 2009). Therefore, Foxp3-mediated suppression of SMS1 results in accumulation of ceramide in Treg cells that leads to activation of the PP2A complex (Apostolidis et al., 2016). PP2A inhibits the mTORC1/AKT pathway in Tregs which is involved in the regulation of cytokine production (IL-2/IL17) and is important for the supressive function of Treg cells (Apostolidis et al., 2016). Treg specific depletion of PP2A led to a multi-organ, lymphoproliferative autoimmune disorder that manifested in mice by the age of 10–14 weeks (Table 1) (Apostolidis et al., 2016).

**TABLE 1:** Animal studies, showing the importance of sphingolipids in T cells.

Sphingolipid class	Animal model	Treatment	T Cell population	References
-	SCID and rag1^-/-^	DSS (colitis)	Adoptive Treg transfer led to resolution of colitis	Mottet et al. (2003)
	Foxp3^YFP-cre^Ppp2r1a^flox/flox^	-	Tregs displayed different metabolic and cytokine profile and did not show immune suppressive activity	Apostolidis et al. (2016)
Sphingomyelins and Ceramides	ASM^-/-^	-	Tregs were elevated and more activated	Hollmann et al. (2016)
Glycosphingolipids	T cell specific Ugcg^-/-^	DSS (colitis)	Treg levels were decreased and the pathogenesis of colitis was more severe compared to control	Komuro et al. (2022)
C22-24 Ceramides	CerS2^-/-^	Ovalbumin (asthma)	TH2 response was impared and Th17 response was elevated accompanied by higher TCR signalling activity	Shin et al. (2021)
Ceramides	CerS6^-/-^	GVHD	T cell response to alloantigen was impaired and differentiation into Th1 cells was inhibited	Sofi et al. (2017)
Ceramides	CerS5^-/-^	DSS (colitis)	CD8^+^ cells were reduced and TCR signalling impaired	El-Hindi et al. (2020)
Ceramides	T cell specific CerS4^-/-^	DSS (colitis)	Prolonged TCR activation in CD8 cells	El-Hindi et al. (2022)

Another mechanism how sphingomyelin influences Tregs is described by Hollmann et al. (Hollmann et al., 2016). They demonstrated that in acid sphingomyelinase (ASMase)-deficient mice a higher number of splenic Tregs could be observed in comparison to control mice (Table1). ASMase is located in endo-lysosomes and catalyzes the hydrolysis of sphingomyeline to phosphorylcholine and ceramide. This enzyme has a pH optimum of 5.0. After certain stimuli ASMase translocates from the lysosome to the plasma membrane which results in the formation of ceramide-enriches membrane platforms. In T cells ASMase is activated after stimulation of CD28 but not after CD3 stimulation (Boucher et al., 1995). In wt mice ASMase activity is higher in Tregs than in CD4^+^/FoxP3^-^/CD25^-^ T cells upon CD28 stimulation. This leads to an increase in ceramide content and higher amount of low lipid order membranes in Tregs. In ASMase deficient Tregs (as well as to a lower amount in CD4+/FoxP3-/CD25- T cells) the percentage of low lipid order membranes is reduced, which goes along with a higher sphingomyelin/ceramide ratio, and facilitates T cell activation (Hollmann et al., 2016). In ASMase knockout Treg cells neither CD25 nor IL-10 were increased in comparison to wt Tregs but the expression of CTLA-4 (Cytotoxic T-Lymphocyte-Associated Protein 4) at the membrane is increased that competes with CD28 for the binding to B7 (CD80/CD86) and additionally removes co-stimulatory ligands from the surface of APCs by transendocytosis (Hollmann et al., 2016). Also in a clinical study it has been shown that treatment of depressive patients with the antidrepessant and acid sphingomyelinase inhibitor sertraline leads to an increase in Tregs among CD4^+^ T cells which was dependent on CD28 activation (Wiese et al., 2021).

Not only sphingomyelin but also glycosylceramide or gangliosides are important for Treg development. The UDP-glucose ceramide glucosyltransferase (UGCG) is the first enzyme in the process of glycosphingolipid biosynthesis. In IBD patients and in a DSS induced colitis mouse model UGCG activity was decreased, and knockdown of UGCG resulted in Treg decrease and CD4 effector cell increase (Table1). In line with this, intravenouse application of nanoparticles loaded with glucosylceramide (GluCer) into DSS treated mice improved colitis symptomes in these mice and enhaced Treg cells in spleen and colon (Komuro et al., 2022). These data indicate that Treg development and function depends on different sphingolipids that impact activation and differentiation of Tregs at various cell stages.

### The role of gangliosides in Th17 development

**FIGURE 4:**
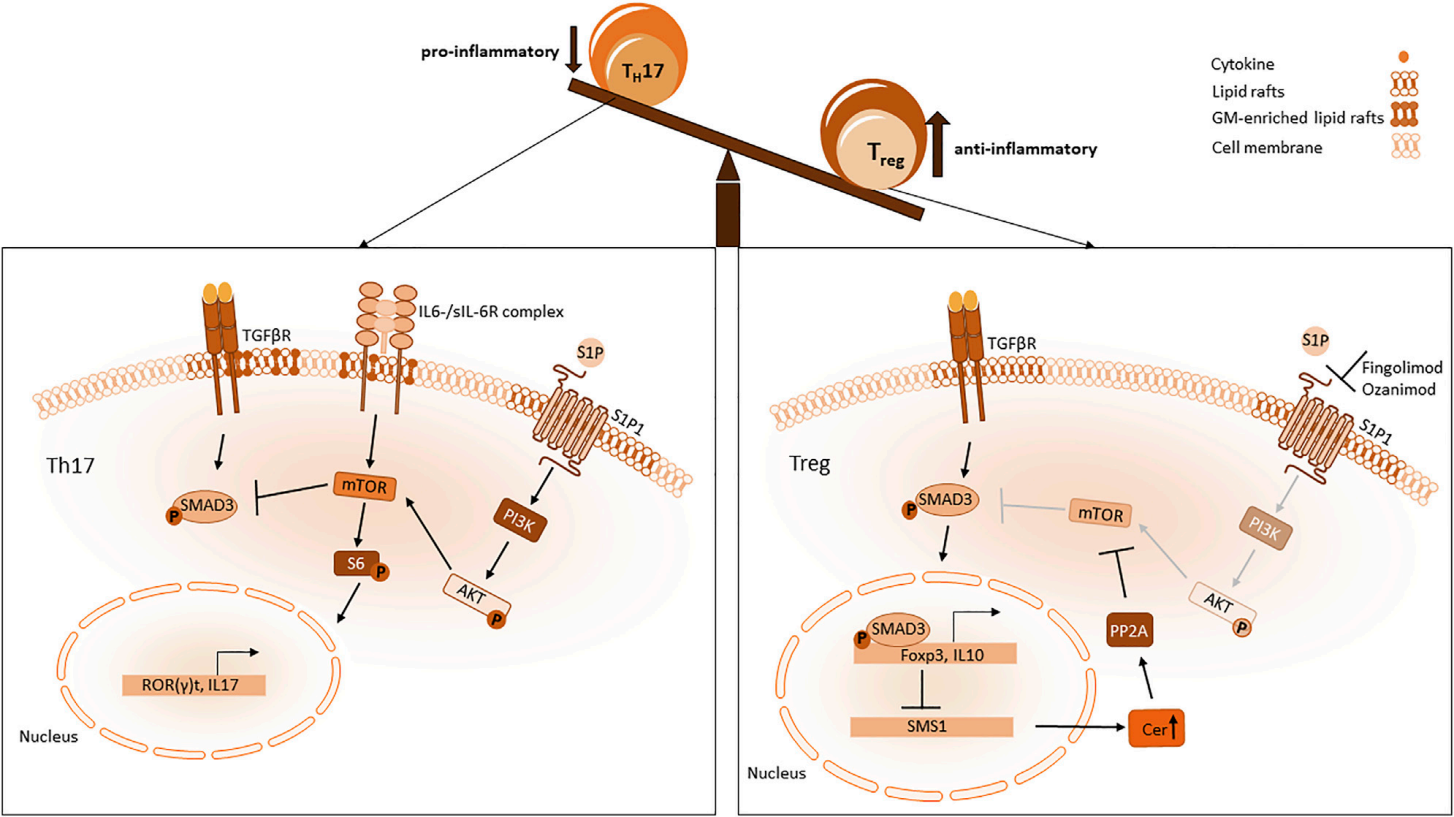
Sphingolipid influence on Th17 and Treg imbalance. In a pro-inflammatory setting Th17 differentiation is mediated through mTOR which is activated by S1P1 and IL6 Receptor signalling. MTOR activates S6 phosphorylation resulting in a Th17 favouring expression pattern with ROR(γ)t and IL-17 expression. Blockade of Smad3 activity mediated via S1P1 dependent mTOR pathway inhibits Foxp3 expression. When S1P1 is antagonized, mTOR signalling is diminished and activity of Smad3 mediated through the TGFβ pathway is sustained. Smad3 enhances Foxp3 expression, which impairs SMS1 transcription resulting in an increased level of ceramides in the cytosol. PP2A interacts with those ceramides and attenuates mTOR signalling.

A dysbalance between Th17 and Treg cells that leans toward the pro-inflammatory Th17 phenotype is also one of the hallmarks of inflammation. To resolve the inflammatory site it should also be considered if pro-inflammatory cells could be inhibited by sphingolipids. It has already been shown that the activation of CD4 and CD8 T cells depends on different gangliosides in their membrane (Nagafuku et al., 2012). Especially during the development of Th17 cells from Th0 cells gangliosides (GMs) and LacCer are upregulated and build a specific signature of differentiated CD4^+^ Th17cells (Sen et al., 2021). The important role of GMs during this differentiation process is supported by the finding that a deficiency of GM3S (GM3 synthase) attenuates the differentiation of CD4^+^ T cells to Th17 cells (Zhu et al., 2011). As GMs are enriched in lipid rafts changes in their expression are likely responsible for alterations of lipid raft associated protein functions. The differentiation of Th17 cells is strongly dependent on the IL-6 receptor (Glycoprotein 130, GP130) and TGF-β (Figure 4). Both receptors have been shown to be located in lipid rafts and transduce their signal *via* the STAT and MAPK pathway (Zuo and Chen, 2009; Allen et al., 2014). Allen et al. have shown that Th17 differentiation is dependent on lipid-raft dependent IL-6 responsiveness (Allen et al., 2014). But also TGFβ receptor signalling and endocytosis is dependent on its localisation into specific lipid rafts at the plasma membrane (Le Roy and Wrana, 2005). Future studies will show if alterations in GM can directly affect IL-6 or TGFβ signalling in Th17 cells via disrupting their association within lipid rafts.

### The impact of ceramides on TCR activity

T cell activation depends on the assembly of different proteins around the TCR in lipid rafts. Cholesterol binds directly to the TCRβ chain facilitating nanoclustering of TCRs independently of antigen binding (Molnár et al., 2012). TCR activation is also affected, when the lipid order is disturbed, for example, by abolishment of neutral SMase 2 (Börtlein et al., 2019). But also ceramides of distinct chain length are important for T cell function. As described above, six mammalian CerS are responsible for the acyl chain length of sphingolipids. CerS2 knockout mice were less sensitive against Ovalbumin-induced allergic asthma which was accompanied by an impaired Th2 response and increased Th17 response. Notably, the increased Th17 response was only visible after TCR stimulation, indicating that loss of very-long chain ceramides in CerS2 knockout mice affects TCR signalling in Th17 cells (Table 1) (Shin et al., 2021). However also CerS4, CerS5 and CerS6 affect TCR signalling, although in different ways. Inhibition of CerS6 in T cells prevents the differentiation into Th1 cells and impairs TCR signal transduction which was related to a reduced co-localization of PKCθ with CD3 at the plasma membrane (Table 1) (Sofi et al., 2017). In CerS5 knockout mice the number of CD3^+^/CD8^+^ and Treg cells was reduced in the intra-epithelial lymphocyte fraction of the colon and TCR signalling was impaired (Table 1) (El-Hindi et al., 2020). Instead, depletion of CerS4 in T cells led to a constitutively and prolonged TCR activation (Table1) (El-Hindi et al., 2022). These data indicate that the molecular composition of the membrane around the TCR is important for proper T cell activation and can be influenced by sphingolipids of various chain length.

### The impact of S1P on T cell migration and signalling

S1P is a bioactive lipid mediator, that is, generated ubiquitously. S1P is formed from ceramide, which is recycled from sphingomyelin by sphingomyelinases and then converted to sphingosine by ceramidases. Two isoenzymes, sphingosine kinase 1 and 2 (SphK), phosphorylate sphingosine to S1P. Both enzymes are located differently, SphK1 can be found in the cytoplasm and the cell membrane, whereas SphK2 is located mainly in mitochondria, nucleus and the ER. S1P is exported out of cells by transporters (such as spinster homolog 2 (SPNS2)) and binds to chaperones in the vascular system (Obinata and Hla, 2019). S1P is known to control many cellular processes via binding and signalling to the G-coupled receptors S1P1-5. S1P and S1P1-5 are crucial for inflammatory responses. Especially S1P_1_ is improtant for lymphocyte trafficking and vascular integrity (Schwab and Cyster, 2007). A gradient of S1P is built between lymph organs, tissues and blood. High concentrations of S1P in the thymus and low concentrations in blood inhibits the egress of T cells and low concentrations in the thymus combined with high concentrations in blood facilitate T cell trafficking out of the thymus. Maintenance of high S1P levels in blood is mediated by SphKs, while low levels of S1P in lymphoid organs is obtained by S1P lyase activity. Binding of S1P to its receptors subsequently leads to an internalisation of the receptors. Co-expression of chemokine receptors can influence S1P receptor 1 surface expression. Expression of CCR7 in T cells, for example, leads T cells into lymphnodes and deletion of CCR7 promotes T cell egress (Debes et al., 2005). Upon TCR stimulation CCR7 expression is downregulated which results in more egress of T cells into the blood. S1P1 is then internalised after S1P exposure and T cells become unresponsive to S1P gradient and migration signals. The internalisation and desensitization of S1P1 is very crucial to the S1P response, incomplete S1PR internalization can promote Th17 dependent autoimmune neuro inflammation (Garris et al., 2013). S1P1 deletion in Th17 cells could bear a resistance to EAE, while deletion in Tregs led to autoimmunity in a MS model (Eken et al., 2017). In inflamed tissue cells respond to Interferon release with the upregulation of CD69, a membrane-bound, type II C-lectin receptor (Cibrián and Sánchez-Madrid, 2017). CD69 binds S1P1 and stabilizes a conformation that mimics S1P1 in a ligand-bound state and induces its internalization, resulting in the trapping of naïve T cells in inflammatory tissue (Shiow et al., 2006; Bankovich et al., 2010). S1P1 also delivers an intrinsic negative feedback loop to decrease thymic production of Tregs thereby suppressing Treg activity via Akt-mTOR pathway (Liu G. et al., 2009). MTOR signalling favours Th17 differentiation and suppresses Treg functions by antagonizing important pathways for Treg activity (Liu et al., 2010). Sustained Smad3 activity is important for Foxp3 expression by TGF-β signalling in Tregs and is antagonized by mTOR pathway activated by S1P1 (Liu et al., 2010). Activation of STAT3 induces Th17 differentiation and attenuates Treg development (Garris et al., 2013). In summary, S1P does not only impact T cell migration but also affects the development of Tregs and Th17 cells, favouring the development of Th17 (Figure 4).

### Enhancing the resolution process by pharmacological interaction with the sphingolipid pathway

As described above several enzymes of the sphingolipid pathway can influence T-cell function and differentiation and might be new targets for the pharmacological treatment of inflammation.

The repurposing of drugs that are already used for a long time in patient treatment is an auspicious approach to accelerate the approval of drugs for new treatment targets. Inhibition of ASMase by tricyclic antidepressants like amitriptyline, fluoxetine or sertraline is one of these examples (Kornhuber et al., 2008). Amitriptyline accumulates in the late lysosomes (low pH) and inhibits the translocation of ASMase from lysosomes to the plasma membrane which is subsequently inactivated by proteolytic degradation (Beckmann et al., 2014). How this leads to an increase in Tregs in human blood, as mentioned above for sertraline (Wiese et al., 2021), might be explained by the molecular mechanisms that are induced by the inhibition of ASMase. Inhibition of ASMase by amitriptyline or fluoxetine leads to an accumulation of sphingomyelin in lysosomes and Golgi membranes and ceramides in the endoplasmic reticulum. This leads to the induction of autophagy in cells (Gulbins et al., 2018). Th17 cells are highly sensitive to autophagy induction which results in a switch of gene expression with lower IL17 expression level and enhanced Foxp3 expression that converts these cells into Tregs. In accordance with that the Treg phenotype is highly dependent on autophagy and inhibition of autophagy in Tregs leads to increased production of IL17A in these cells (Park et al., 2016; González-Osuna et al., 2022). These data indicate that the induction of autophagy in Th17 cells might be a trigger to switch the Th17 phenotype to a Treg phenotype thereby promoting anti-inflammatory mechanisms that contribute to the resolution of inflammation. Data from animal studies of cystic fibrosis as well as a clinical phase IIb study indicate that amitriptyline improves lung inflammation and function (Table 2), which might be related to the functional switch of T cells (Becker et al., 2010; Nährlich et al., 2013). These data indicate that tricyclic antidepressants might be new treatment option for chronic inflammatory diseases that are characterized by an unbalance between Th17 and Treg cells.

**TABLE 2:** Ongoing clinical trials with substances that interfere with the sphingolipid pathway.

Substance	Target	Indication	Clinical phase	Clinical trial No	Status
Amitriptyline	ASMase	Lung inflammation	II	NCT00515229	completed
Venclustat	GCS	Sandhoff disease (Late-onset GM2)	III	NCT04221451	a.n. recruiting
Venclustat	GCS	Fabry’s disease	III	NCT05206773	recruiting
			III	NCT05280548	recruiting
Venclustat	GCS	Gaucher’s Disease Type III	III	NCT05222906	recruiting
Venclustat	GCS	Gaucher Disease Type 1/3	II	NCT02843035	a.n. recruiting
Fingolimod, Ofatumumab, Siponimod	S1P_1_, S1P_3_, S1P_4_, S1P_5_ CD20, S1P_1_, S1P_5_	Multiple sclerosis	III	NCT04926818	recruiting
Etrasimod	S1P_1_, S1P_4_, S1P_5_	Ulcerative colitis	II	NCT04607837	a.n. recruiting
			II	NCT05061446	recruiting
			II	NCT05287126	recruiting
			III	NCT04706793	a.n. recruiting
			III	NCT04176588	recruiting
Etrasimod	S1P_1_, S1P_4_, S1P_5_	Eosinophilic esophagitis	II	NCT04682639	a.n.recruiting
Etrasimod	S1P_1_, S1P_4_, S1P_5_	Crohn’s disease	II/III	NCT04173273	recruiting
Ponesimod	S1P_1_, S1P_4_, S1P_5_	Multiple sclerosis	III	NCT03232073	a.n. recruiting
			II	NCT01093326	a.n. recruiting
			n.s.	NCT03500328	recruiting
			IV	NCT03535298	recruiting
Amiselimod	S1P_1_	Colitis	II	NCT04857112	recruiting
Ozanimod	S1P_1_, S1P_5_	Colitis	II/III	NCT05076175	recruiting
			III	NCT03915769	recruiting
Ozanimod	S1P_1_, S1P_5_	Crohn’s disease	II/III	NCT05470985	not yet recruiting
			III	NCT03467958	recruiting
Ozanimod	S1P_1_, S1P_5_	Multiple sclerosis	n.s	NCT05245344	not yet recruiting
				NCT05028634	recruiting
Siponimod	S1P_1_, S1P_5_	Multiple sclerosis	n.s	NCT04895202	recruiting
			n.s	NCT05376579	recruiting
			IV	NCT04792567	a.n. recruiting
			IV	NCT04925557	recruiting

ns = not specified.

a.n.recruiting = active not recruiting.

Although, GM3S seems to be an additional beneficial target to manipulate the equilibrium between Th17 and Tregs, unfortunately inhibition of this enzyme might have severe side effects as mutations in the ST3GAL5 gene (encodes for GM3S) are associated with severe infantile-onset of neurological symptoms such as progressive microcephaly, intellectual disability, dyskinetic movements, blindness and deafness (Inamori and Inokuchi, 2022). Gangliosides are important lipid components of the myelin sheath in the central and peripheral nervous system. So, the application of substances that inhibit GM3S should be cell type specific. As from right now, no specific GM3S inhibitor is on the market (Yamanaka et al., 2020). However, a glycosyl-ceramide synthase (GCS) inhibitor might be a useful alternative. Venglustat is an inhibitor of GCS and originally developed for the treatment of patients with lysosomal storage disease (Gaucher disease type 3, Fabry disease and GM2 Gangliosidosis, Sandhoff disease, see also clinical trials in Table 2). GCS couples the first glycoside to ceramide resulting in GlyCer, that is, the precursor for all other complex gangliosides, as well as for the GM3S. Fabry patients suffer additionally from airway inflammation. Treatment of these patients with an enzyme replacement therapy using recombinant human α-galactosidase A enzyme, which partial restores the glycosphingolipid level, stabilizes obstructive lung disease and decreased Th17 cells while increase Th1 cells (Odler et al., 2017). As this was only a very small study, further data are needed. The impact of venglustat on the development of Th17 or Tregs has not been investigated in detail so far.

Furthermost progress is made in the pharmacological inhibition of the S1P signaling pathway. Fingolimod (FTY720) is a prodrug, that is, phosphorylated by Sphk2 to its active metabolite fingolimod-phosphate. It is an unselective S1P receptor agonist, with exception of S1P_2,_ and after binding to S1P_1_ it leads to an internalization and degradation of this receptor. Fingolimod has immunomodulatory effects and is approved for the treatment of multiple sclerosis patients since 2011 (Stepanovska and Huwiler, 2020). Although, Fingolimod shows promising data in animal studies for ulcerative colitis (UC) (Makled et al., 2022), it is not approved for this indication. Instead, Ozanimod, a selective S1P receptor modulator with high affinity to S1P1 and S1P5 is approved for UC patients. A phase III clinical trial shows a significantly higher clinical remission in colitis patients after ozanimod treatment in comparison to placebo treatment (Sandborn et al., 2021). Other selective S1P receptor modulators are in development or used in clinical trials like Etrasimod (selective modulator of S1PR1,4,5, phase III clinical trial in UC patients), Amiselimod (high affinity to S1PR1, clinical trials in Crohn’s disease (CD)), Ponesimod (selective modulator of S1PR1,4,5 tested in clinical trial phase II for psoriasis) (Pérez-Jeldres et al., 2021) (Table 2). FTY720-p, siponimod, etrasimod, ponesimod, ozanimod, and amiselimod-p bind to the orthosteric site of S1P_1_ and S1P_5_. They display similar receptor pharmacology and compete for binding at the same site. Therefore, they can be considered interchangeable with one another (Selkirk et al., 2022). Furthermore, current clinical trials compare the safety and efficacy of combination therapy combining fingolimod, siponimod and ofatumumab (anti-CD20 antibody) in pediatric patients with multiple sclerosis (Table 2). A very comprehensive overview about S1PR modulators in clinical studies is given in (Stepanovska and Huwiler 2020).

These data indicate, that the pharmacological interaction with S1P signaling to influence T cell migration or function is a promising approach to treat inflammatory conditions.

## Conclusion

The resolution of inflammation gained a lot of attention in the past few years as an active process of re-establishing tissue-homeostasis and resolving inflammatory processes. Tregs regulate macrophage responses, immunosuppressive cytokine release and restoration of tissue homeostasis whereas pro-inflammatory Th1/2/17 cells promote inflammation. In this review we highlighted how sphingolipids can interfere with the differentiation and activation process of T cells. A particular sphingolipid composition of the membrane seems to be required for the different subsets of T cells and the formation of variable immunological synapses. For this reason sphingolipids influence diverse chronic inflammatory diseases that are driven by Th1 or Th2 cell response, and an imbalance between Th17 and Tregs such as in rheumatoid arthritis (Tsukuda et al., 2012) or IBD patients (Komuro et al., 2022) and are important for the resolution of inflammation. So, in future studies enzymes of the sphingolipid pathway as well as sphingolipids themselves should be considered as new possible targets to boost resolving processes and to dampen pro-inflammatory mechanisms. Ongoing clinical trials with various sphingolipid modulators might shed light on the utility of these substances in different inflammatory diseases. However, to proof if anti-inflammatory effects are mediated by the impact of these substances on T cells, future studies should also investigate the T cell subtypes in blood after treatment of patients with sphingolipid modulators. Additionally, investigating the effect of sphingolipid inhibitors on primary human blood cells *in vitro* using lipidomics, transcriptomics and characterizing released cytokines by cytometric bead array assay (CBA), might give a hint how these substances influence T cell subtypes and which signaling pathways are involved in the resolution of inflammation.

## References

[B1] Allen M. J. Fan Y-Y. Monk J. M. Hou T. Y. Barhoumi R. McMurray D. N. (2014). n-3 PUFAs reduce T-helper 17 cell differentiation by decreasing responsiveness to interleukin-6 in isolated mouse splenic CD4⁺ T cells. J. Nutr. 144, 1306–1313. 10.3945/jn.114.194407 24944284 PMC4093987

[B2] Aouad S. M. Cohen L. Y. Sharif-Askari E. Haddad E. K. Alam A. Sekaly R-P. (2004). Caspase-3 is a component of Fas death-inducing signaling complex in lipid rafts and its activity is required for complete caspase-8 activation during Fas-mediated cell death. J. Immunol. 172, 2316–2323. 10.4049/jimmunol.172.4.2316 14764700

[B3] Apostolidis S. A. Rodríguez-Rodríguez N. Suárez-Fueyo A. Dioufa N. Ozcan E. Crispín J. C. (2016). Phosphatase PP2A is requisite for the function of regulatory T cells. Nat. Immunol. 17, 556–564. 10.1038/ni.3390 26974206 PMC4837024

[B4] Artham S. Verma A. Alwhaibi A. Adil M. S. Manicassamy S. Munn D. H. (2020). Delayed Akt suppression in the lipopolysaccharide-induced acute lung injury promotes resolution that is associated with enhanced effector regulatory T cells. Am. J. Physiol. Lung Cell Mol. Physiol. 318, L750–L761. 10.1152/ajplung.00251.2019 32073894 PMC7191478

[B5] Artyomov M. N. Lis M. Devadas S. Davis M. M. Chakraborty A. K. (2010). CD4 and CD8 binding to MHC molecules primarily acts to enhance Lck delivery. Proc. Natl. Acad. Sci. U. S. A. 107, 16916–16921. 10.1073/pnas.1010568107 20837541 PMC2947881

[B6] Balagopalan L. Kortum R. L. Coussens N. P. Barr V. A. Samelson L. E. (2015). The linker for activation of T cells (LAT) signaling hub: From signaling complexes to microclusters. J. Biol. Chem. 290, 26422–26429. 10.1074/jbc.R115.665869 26354432 PMC4646300

[B7] Bankovich A. J. Shiow L. R. Cyster J. G. (2010). CD69 suppresses sphingosine 1-phosophate receptor-1 (S1P1) function through interaction with membrane helix 4. J. Biol. Chem. 285, 22328–22337. 10.1074/jbc.M110.123299 20463015 PMC2903414

[B8] Becker K. A. Riethmüller J. Lüth A. Döring G. Kleuser B. Gulbins E. (2010). Acid sphingomyelinase inhibitors normalize pulmonary ceramide and inflammation in cystic fibrosis. Am. J. Respir. Cell Mol. Biol. 42, 716–724. 10.1165/rcmb.2009-0174OC 19635928

[B9] Beckmann N. Sharma D. Gulbins E. Becker K. A. Edelmann B. (2014). Inhibition of acid sphingomyelinase by tricyclic antidepressants and analogons. Front. Physiol. 5, 331. 10.3389/fphys.2014.00331 25228885 PMC4151525

[B10] Bieberich E. (2018). Sphingolipids and lipid rafts: Novel concepts and methods of analysis. Chem. Phys. Lipids 216, 114–131. 10.1016/j.chemphyslip.2018.08.003 30194926 PMC6196108

[B11] Birnbaum M. E. Berry R. Hsiao Y-S. Chen Z. Shingu-Vazquez M. A. Yu X. (2014). Molecular architecture of the αβ T cell receptor-CD3 complex. Proc. Natl. Acad. Sci. U. S. A. 111, 17576–17581. 10.1073/pnas.1420936111 25422432 PMC4267357

[B12] Börtlein C. Schumacher F. Kleuser B. Dölken L. Avota E. (2019). Role of neutral sphingomyelinase-2 (NSM 2) in the control of T cell plasma membrane lipid composition and cholesterol homeostasis. Front. Cell Dev. Biol. 7, 226. 10.3389/fcell.2019.00226 31681760 PMC6803391

[B13] Boucher L-M. Wiegmann K. Fütterer A. Pfeffer K. Machleidt T. Schütze S. (1995). CD28 signals through acidic sphingomyelinase. J. Exp. Med. 181, 2059–6810. 10.1084/jem.181.6.2059 7759998 PMC2192051

[B14] Bradley P. Thomas P. G. (2019). Using T cell receptor repertoires to understand the principles of adaptive immune recognition. Annu. Rev. Immunol. 37, 547–570. 10.1146/annurev-immunol-042718-041757 30699000

[B15] Brembilla N. C. Montanari E. Truchetet M-E. Raschi E. Meroni P. Chizzolini C. (2013). Th17 cells favor inflammatory responses while inhibiting type I collagen deposition by dermal fibroblasts: Differential effects in healthy and systemic sclerosis fibroblasts. Arthritis Res. Ther. 15, R151. 10.1186/ar4334 24289089 PMC3979123

[B16] Brown S. B. Savill J. (1999). Phagocytosis triggers macrophage release of Fas ligand and induces apoptosis of bystander leukocytes. J. Immunol. 162, 480–485. 9886423

[B17] Buchholz V. R. Schumacher T. N. M. Busch D. H. (2016). T cell fate at the single-cell level. Annu. Rev. Immunol. 34, 65–92. 10.1146/annurev-immunol-032414-112014 26666651

[B18] Caja L. Dituri F. Mancarella S. Caballero-Diaz D. Moustakas A. Giannelli G. (2018). TGF-Β and the tissue microenvironment: Relevance in fibrosis and cancer. Int. J. Mol. Sci. 19. 10.3390/ijms19051294 PMC5983604 29701666

[B19] Capolupo L. Khven I. Lederer A. R. Mazzeo L. Glousker G. Ho S. (2022). Sphingolipids control dermal fibroblast heterogeneity. Science 376, eabh1623. 10.1126/science.abh1623 35420948

[B20] Carding S. R. Egan P. J. (2002). Gammadelta T cells: Functional plasticity and heterogeneity. Nat. Rev. Immunol. 2, 336–345. 10.1038/nri797 12033739

[B21] Cerbón J. Baranda-Avila N. Falcón-Muñoz A. Camacho-Arroyo I. Cerbón M. (2018). Sphingolipid synthesis and role in uterine epithelia proliferation. Reproduction 156, 173–183. 30054445 10.1530/REP-17-0667

[B22] Chongsathidkiet P. Jackson C. Koyama S. Loebel F. Cui X. Farber S. H. (2018). Sequestration of T cells in bone marrow in the setting of glioblastoma and other intracranial tumors. Nat. Med. 24, 1459–1468. 10.1038/s41591-018-0135-2 30104766 PMC6129206

[B23] Cibrián D. Sánchez-Madrid F. (2017). CD69: From activation marker to metabolic gatekeeper. Eur. J. Immunol. 47, 946–953. 10.1002/eji.201646837 28475283 PMC6485631

[B24] Cox M. A. Zajac A. J. (2010). Shaping successful and unsuccessful CD8 T cell responses following infection. J. Biomed. Biotechnol. 2010, 159152. 10.1155/2010/159152 20379363 PMC2850140

[B25] Croker B. A. O'Donnell J. A. Nowell C. J. Metcalf D. Dewson G. Campbell K. J. (2011). Fas-mediated neutrophil apoptosis is accelerated by bid, bak, and bax and inhibited by bcl-2 and mcl-1. Proc. Natl. Acad. Sci. U. S. A. 108, 13135–13140. 10.1073/pnas.1110358108 21768356 PMC3156212

[B26] Debes G. F. Arnold C. N. Young A. J. Krautwald S. Lipp M. Hay J. B. (2005). Chemokine receptor CCR7 required for T lymphocyte exit from peripheral tissues. Nat. Immunol. 6, 889–9410. 10.1038/ni1238 16116468 PMC2144916

[B27] Eken A. Duhen R. Singh A. K. Fry M. Buckner J. H. Kita M. (2017). S1P1 deletion differentially affects TH17 and Regulatory T cells. Sci. Rep. 7, 12905. 10.1038/s41598-017-13376-2 29018225 PMC5635040

[B28] El-Hindi K. Brachtendorf S. Hartel J. C. Oertel S. Birod K. Merz N. (2022). T-Cell-Specific CerS4 depletion prolonged inflammation and enhanced tumor burden in the AOM/DSS-Induced CAC model. Int. J. Mol. Sci. 23. 10.3390/ijms23031866 PMC8837088 35163788

[B29] El-Hindi K. Brachtendorf S. Hartel J. C. Oertel S. Birod K. Trautmann S. (2020). Ceramide synthase 5 deficiency aggravates dextran sodium sulfate-induced colitis and colon carcinogenesis and impairs T-cell activation. Cancers (Basel) 12. 10.3390/cancers12071753 PMC7409364 32630271

[B30] Eppensteiner J. Kwun J. Scheuermann U. Barbas A. Limkakeng A. T. Kuchibhatla M. (2019). Damage- and pathogen-associated molecular patterns play differential roles in late mortality after critical illness. JCI Insight 4. 10.1172/jci.insight.127925 PMC6777836 31434802

[B31] Falasca M. Logan S. K. Lehto V. P. Baccante G. Lemmon M. A. Schlessinger J. (1998). Activation of phospholipase C gamma by PI 3-kinase-induced PH domain-mediated membrane targeting. EMBO J. 17, 414–2210. 10.1093/emboj/17.2.414 9430633 PMC1170392

[B32] Fang P. Li X. Dai J. Cole L. Camacho J. A. Zhang Y. (2018). Immune cell subset differentiation and tissue inflammation. J. Hematol. Oncol. 11, 97. 10.1186/s13045-018-0637-x 30064449 PMC6069866

[B33] Fife B. T. Bluestone J. A. (2008). Control of peripheral T-cell tolerance and autoimmunity via the CTLA-4 and PD-1 pathways. Immunol. Rev. 224, 166–182. 10.1111/j.1600-065X.2008.00662.x 18759926

[B34] Forthal D. M. (2014). Functions of antibodies. Microbiol. Spectr. 2, AID–201410. 10.1128/microbiolspec.AID-0019-2014 26104200

[B35] Garçon F. Patton D. T. Emery J. L. Hirsch E. Rottapel R. Sasaki T. (2008). CD28 provides T-cell costimulation and enhances PI3K activity at the immune synapse independently of its capacity to interact with the p85/p110 heterodimer. blood 111, 1464–1471. 10.1182/blood-2007-08-108050 18006698

[B36] Garris C. S. Wu L. Acharya S. Arac A. Blaho V. A. Huang Y. (2013). Defective sphingosine 1-phosphate receptor 1 (S1P1) phosphorylation exacerbates TH17-mediated autoimmune neuroinflammation. Nat. Immunol. 14, 1166–1172. 10.1038/ni.2730 24076635 PMC4014310

[B37] González-Osuna L. Sierra-Cristancho A. Cafferata E. A. Melgar-Rodríguez S. Rojas C. Carvajal P. (2022). Senescent CD4+CD28− T lymphocytes as a potential driver of Th17/treg imbalance and alveolar bone resorption during periodontitis. Ijms 23, 2543. 10.3390/ijms23052543 35269683 PMC8910032

[B38] Greenlee-Wacker M. C. (2016). Clearance of apoptotic neutrophils and resolution of inflammation. Immunol. Rev. 273, 357–370. 10.1111/imr.12453 27558346 PMC5000862

[B39] Grösch S. Schiffmann S. Geisslinger G. (2012). Chain length-specific properties of ceramides. Prog. Lipid Res. 51, 50–62. 10.1016/j.plipres.2011.11.001 22133871

[B40] Gulbins A. Schumacher F. Becker K. A. Wilker B. Soddemann M. Boldrin F. (2018). Antidepressants act by inducing autophagy controlled by sphingomyelin-ceramide. Mol. Psychiatry 23, 2324–2346. 10.1038/s41380-018-0090-9 30038230 PMC6294742

[B41] Hadas Y. Vincek A. S. Youssef E. Żak M. M. Chepurko E. Sultana N. (2020). Altering sphingolipid metabolism attenuates cell death and inflammatory response after myocardial infarction. Circulation 141, 916–930. 10.1161/CIRCULATIONAHA.119.041882 31992066 PMC7135928

[B42] Hillion S. Arleevskaya M. I. Blanco P. Bordron A. Brooks W. H. Cesbron J. Y. (2020). The innate part of the adaptive immune system. Clin. Rev. Allergy Immunol. 58, 151–154. 10.1007/s12016-019-08740-1 31154567

[B43] Hollmann C. Werner S. Avota E. Reuter D. Japtok L. Kleuser B. (2016). Inhibition of acid sphingomyelinase allows for selective targeting of CD4+ conventional versus Foxp3+ regulatory T cells. J. Immunol. 197, 3130–3141. 10.4049/jimmunol.1600691 27638864

[B44] Hünig T. Lühder F. Elflein K. Gogishvili T. Fröhlich M. Guler R. (2010). CD28 and IL-4: Two heavyweights controlling the balance between immunity and inflammation. Med. Microbiol. Immunol. 199, 239–246. 10.1007/s00430-010-0156-z 20390297 PMC3128750

[B45] Hwang E. S. Hong J-H. Glimcher L. H. (2005). IL-2 production in developing Th1 cells is regulated by heterodimerization of RelA and T-bet and requires T-bet serine residue 508. J. Exp. Med. 202, 1289–1300. 10.1084/jem.20051044 16275766 PMC2213245

[B46] Imbratta C. Hussein H. Andris F. Verdeil G. (2020). c-MAF, a Swiss army knife for tolerance in lymphocytes. Front. Immunol. 11, 206. 10.3389/fimmu.2020.00206 32117317 PMC7033575

[B47] Inamori K-I. Inokuchi J-i. (2022). Ganglioside GM3 synthase deficiency in mouse models and human patients. Int. J. Mol. Sci. 23. 10.3390/ijms23105368 PMC9141422 35628171

[B48] Jakobi K. Beyer S. Koch A. Thomas D. Schwalm S. Zeuzem S. (2020). Sorafenib treatment and modulation of the sphingolipid pathway affect proliferation and viability of hepatocellular carcinoma *in vitro* . Int. J. Mol. Sci. 21. 10.3390/ijms21072409 PMC7177910 32244391

[B49] Kanhere A. Hertweck A. Bhatia U. Gökmen M. R. Perucha E. Jackson I. (2012). T-bet and GATA3 orchestrate Th1 and Th2 differentiation through lineage-specific targeting of distal regulatory elements. Nat. Commun. 3, 1268. 10.1038/ncomms2260 23232398 PMC3535338

[B50] Kawai T. Akira S. (2007). Signaling to NF-kappaB by toll-like receptors. Trends Mol. Med. 13, 460–469. 10.1016/j.molmed.2007.09.002 18029230

[B51] Kisuya J. Chemtai A. Raballah E. Keter A. Ouma C. (2019). The diagnostic accuracy of Th1 (IFN-γ, TNF-α, and IL-2) and Th2 (IL-4, IL-6 and IL-10) cytokines response in AFB microscopy smear negative PTB- HIV co-infected patients. Sci. Rep. 9, 2966. 10.1038/s41598-019-39048-x 30814543 PMC6393479

[B52] Klein L. Kyewski B. Allen P. M. Hogquist K. A. (2014). Positive and negative selection of the T cell repertoire: What thymocytes see (and don't see). Nat. Rev. Immunol. 14, 377–391. 10.1038/nri3667 24830344 PMC4757912

[B53] Kmieciak M. Gowda M. Graham L. Godder K. Bear H. D. Marincola F. M. (2009). Human T cells express CD25 and Foxp3 upon activation and exhibit effector/memory phenotypes without any regulatory/suppressor function. J. Transl. Med. 7, 89. 10.1186/1479-5876-7-89 19849846 PMC2770477

[B54] Kohno K. Koya-Miyata S. Harashima A. Tsukuda T. Katakami M. Ariyasu T. (2021). Inflammatory M1-like macrophages polarized by NK-4 undergo enhanced phenotypic switching to an anti-inflammatory M2-like phenotype upon co-culture with apoptotic cells. J. Inflamm. 18, 2. 10.1186/s12950-020-00267-z PMC7791770 33413430

[B55] Komuro M. Nagane M. Endo R. Nakamura T. Miyamoto T. Niwa C. (2022). Glucosylceramide in T cells regulates the pathology of inflammatory bowel disease. Biochem. Biophys. Res. Commun. 599, 24–30. 10.1016/j.bbrc.2022.02.004 35168060

[B56] Kornhuber J. Tripal P. Reichel M. Terfloth L. Bleich S. Wiltfang J. (2008). Identification of new functional inhibitors of acid sphingomyelinase using a structure-property-activity relation model. J. Med. Chem. 51, 219–237. 10.1021/jm070524a 18027916

[B57] Kourtzelis I. Hajishengallis G. Chavakis T. (2020). Phagocytosis of apoptotic cells in resolution of inflammation. Front. Immunol. 11, 553. 10.3389/fimmu.2020.00553 32296442 PMC7137555

[B58] Kratky W. Reis e Sousa C. Oxenius A. Spörri R. (2011). Direct activation of antigen-presenting cells is required for CD8+ T-cell priming and tumor vaccination. Proc. Natl. Acad. Sci. U. S. A. 108, 17414–17419. 10.1073/pnas.1108945108 21987815 PMC3198339

[B59] Lawrence T. (2009). The nuclear factor NF-kappaB pathway in inflammation. Cold Spring Harb. Perspect. Biol. 1, a001651. 10.1101/cshperspect.a001651 20457564 PMC2882124

[B60] Le Roy C. Wrana J. L. (2005). Clathrin- and non-clathrin-mediated endocytic regulation of cell signalling. Nat. Rev. Mol. Cell Biol. 6, 112–126. 10.1038/nrm1571 15687999

[B61] Leahy D. J. (1995). A structural view of CD4 and CD8. FASEB J. 9, 17–25. 10.1096/fasebj.9.1.7821755 7821755

[B62] Li J. Hsu H-C. Mountz J. D. (2013a). The dynamic duo-inflammatory M1 macrophages and Th17 cells in rheumatic diseases. J. Orthop. Rheumatol. 1, 4. 10.13188/2334-2846.1000002 25309946 PMC4193941

[B63] Li W. Sandhoff R. Kono M. Zerfas P. Hoffmann V. Ding B. C-H. (2007). Depletion of ceramides with very long chain fatty acids causes defective skin permeability barrier function, and neonatal lethality in ELOVL4 deficient mice. Int. J. Biol. Sci. 3, 120–128. 10.7150/ijbs.3.120 17311087 PMC1796950

[B64] Li Y. Yin Y. Mariuzza R. A. (2013b). Structural and biophysical insights into the role of CD4 and CD8 in T cell activation. Front. Immunol. 4, 206. 10.3389/fimmu.2013.00206 23885256 PMC3717711

[B65] Liao Y. Li G. Zhang X. Huang W. Xie D. Dai G. (2020). Cardiac Nestin+ mesenchymal stromal cells enhance healing of ischemic heart through periostin-mediated M2 macrophage polarization. Mol. Ther. 28, 855–873. 10.1016/j.ymthe.2020.01.011 31991111 PMC7054724

[B66] Lingwood D. Simons K. (2010). Lipid rafts as a membrane-organizing principle. Science 327, 46–50. 10.1126/science.1174621 20044567

[B67] Linsley P. S. Ledbetter J. A. (1993). The role of the CD28 receptor during T cell responses to antigen. Annu. Rev. Immunol. 11, 191–212. 10.1146/annurev.iy.11.040193.001203 8386518

[B68] Liu G. Burns S. Huang G. Boyd K. Proia R. L. Flavell R. A. (2009a). The receptor S1P1 overrides regulatory T cell-mediated immune suppression through Akt-mTOR. Nat. Immunol. 10, 769–777. 10.1038/ni.1743 19483717 PMC2732340

[B69] Liu G. Yang K. Burns S. Shrestha S. Chi H. (2010). The S1P(1)-mTOR axis directs the reciprocal differentiation of T(H)1 and T(reg) cells. Nat. Immunol. 11, 1047–1056. 10.1038/ni.1939 20852647 PMC2958252

[B70] Liu W. Ouyang X. Yang J. Liu J. Li Q. Gu Y. (2009b). AP-1 activated by toll-like receptors regulates expression of IL-23 p19. J. Biol. Chem. 284, 24006–24016. 10.1074/jbc.M109.025528 19592489 PMC2781995

[B71] Lo W-L. Shah N. H. Ahsan N. Horkova V. Stepanek O. Salomon A. R. (2018). Lck promotes Zap70-dependent LAT phosphorylation by bridging Zap70 to LAT. Nat. Immunol. 19, 733–741. 10.1038/s41590-018-0131-1 29915297 PMC6202249

[B72] Loberto N. Mancini G. Bassi R. Carsana E. V. Tamanini A. Pedemonte N. (2020). Sphingolipids and plasma membrane hydrolases in human primary bronchial cells during differentiation and their altered patterns in cystic fibrosis. Glycoconj J. 37, 623–633. 10.1007/s10719-020-09935-x 32666337 PMC7501107

[B73] Makled M. N. Serrya M. S. El-Sheakh A. R. (2022). Fingolimod ameliorates acetic acid-induced ulcerative colitis: An insight into its modulatory impact on pro/anti-inflammatory cytokines and AKT/mTOR signalling. Basic Clin. Pharmacol. Toxicol. 130, 569–580. 10.1111/bcpt.13720 35274449

[B74] McKenzie G. J. Bancroft A. Grencis R. K. McKenzie A. N. J. (1998). A distinct role for interleukin-13 in Th2-cell-mediated immune responses. Curr. Biol. 8, 339–4210. 10.1016/s0960-9822(98)70134-4 9512421

[B75] Merle N. S. Church S. E. Fremeaux-Bacchi V. Roumenina L. T. (2015). Complement system Part I - molecular mechanisms of activation and regulation. Front. Immunol. 6, 262. 10.3389/fimmu.2015.00262 26082779 PMC4451739

[B76] Mignard V. Dubois N. Lanoé D. Joalland M-P. Oliver L. Pecqueur C. (2020). Sphingolipid distribution at mitochondria-associated membranes (MAMs) upon induction of apoptosis. J. Lipid Res. 61, 1025–1037. 10.1194/jlr.RA120000628 32350079 PMC7328052

[B77] Moens U. Kostenko S. Sveinbjørnsson B. (2013). The role of mitogen-activated protein kinase-activated protein kinases (MAPKAPKs) in inflammation. Genes (Basel) 4, 101–133. 10.3390/genes4020101 24705157 PMC3899974

[B78] Molnár E. Swamy M. Holzer M. Beck-García K. Worch R. Thiele C. (2012). Cholesterol and sphingomyelin drive ligand-independent T-cell antigen receptor nanoclustering. J. Biol. Chem. 287, 42664–42674. 10.1074/jbc.M112.386045 23091059 PMC3522267

[B79] Monasterio B. G. Jiménez-Rojo N. García-Arribas A. B. Riezman H. Goñi F. M. Alonso A. (2021). CHO/LY-B cell growth under limiting sphingolipid supply: Correlation between lipid composition and biophysical properties of sphingolipid-restricted cell membranes. FASEB J. 35, e21657. 10.1096/fj.202001879RR 34010474 PMC12315980

[B80] Mottet C. Uhlig H. H. Powrie F. (2003). Cutting edge: Cure of colitis by CD4+CD25+ regulatory T cells. J. Immunol. 170, 3939–3943. 10.4049/jimmunol.170.8.3939 12682220

[B81] Muench D. E. Sun Z. Sharma A. Tang C. Crampton J. S. Lao C. (2022). A pathogenic Th17/cd38+ macrophage feedback loop drives inflammatory arthritis through TNF-α. J. Immunol. 208, 1315–1328. 10.4049/jimmunol.2101025 35197330

[B82] Mukhopadhyay A. Saddoughi S. A. Song P. Sultan I. Ponnusamy S. Senkal C. E. (2009). Direct interaction between the inhibitor 2 and ceramide via sphingolipid-protein binding is involved in the regulation of protein phosphatase 2A activity and signaling. FASEB J. 23, 751–763. 10.1096/fj.08-120550 19028839 PMC2653988

[B83] Murao A. Aziz M. Wang H. Brenner M. Wang P. (2021). Release mechanisms of major DAMPs. Apoptosis 26, 152–162. 10.1007/s10495-021-01663-3 33713214 PMC8016797

[B84] Muthusamy T. Cordes T. Handzlik M. K. You Le Lim E. W. Gengatharan J. (2020) Serine restriction alters sphingolipid diversity to constrain tumour growth. Nature 586:790–795. 10.1038/s41586-020-2609-x 32788725 PMC7606299

[B85] Nagafuku M. Okuyama K. Onimaru Y. Suzuki A. Odagiri Y. Yamashita T. (2012). CD4 and CD8 T cells require different membrane gangliosides for activation. Proc. Natl. Acad. Sci. U. S. A. 109, E336–E342. 10.1073/pnas.1114965109 22308377 PMC3277553

[B86] Nährlich L. Mainz J. G. Adams C. Engel C. Herrmann G. Icheva V. (2013). Therapy of CF-patients with amitriptyline and placebo--a randomised, double-blind, placebo-controlled phase IIb multicenter, cohort-study. Cell Physiol. Biochem. 31, 505–512. 10.1159/000350071 23572075

[B87] Nanno M. Shiohara T. Yamamoto H. Kawakami K. Ishikawa H. (2007). Γδ T cells: Firefighters or fire boosters in the front lines of inflammatory responses. Immunol. Rev. 215, 103–113. 10.1111/j.1600-065X.2006.00474.x 17291282

[B88] Ng T. H. S. Britton G. J. Hill E. V. Verhagen J. Burton B. R. Wraith D. C. (2013). Regulation of adaptive immunity; the role of interleukin-10. Front. Immunol. 4, 129. 10.3389/fimmu.2013.00129 23755052 PMC3668291

[B89] Obinata H. Hla T. (2019). Sphingosine 1-phosphate and inflammation. Int. Immunol. 31, 617–625. 10.1093/intimm/dxz037 31049553 PMC6939830

[B90] Odler B. Cseh Á. Constantin T. Fekete G. Losonczy G. Tamási L. (2017). Long time enzyme replacement therapy stabilizes obstructive lung disease and alters peripheral immune cell subsets in Fabry patients. Clin. Respir. J. 11, 942–950. 10.1111/crj.12446 26763180

[B91] Orecchioni M. Ghosheh Y. Pramod A. B. Ley K. (2019). Macrophage polarization: Different gene signatures in M1(LPS+) vs. Classically and M2(LPS-) vs. Alternatively activated macrophages. Front. Immunol. 10, 1084. 10.3389/fimmu.2019.01084 31178859 PMC6543837

[B92] Osińska I. Popko K. Demkow U. (2014). Perforin: An important player in immune response. Cent. Eur. J. Immunol. 39, 109–115. 10.5114/ceji.2014.42135 26155110 PMC4439970

[B93] Pałgan K. Bartuzi Z. (2015). Platelet activating factor in allergies. Int. J. Immunopathol. Pharmacol. 28, 584–589. 10.1177/0394632015600598 26486136

[B94] Palmer E. (2003). Negative selection--clearing out the bad apples from the T-cell repertoire. Nat. Rev. Immunol. 3, 383–391. 10.1038/nri1085 12766760

[B95] Park M-J. Lee S-Y. Moon S-J. Son H-J. Lee S-H. Kim E-K. (2016). Metformin attenuates graft-versus-host disease via restricting mammalian target of rapamycin/signal transducer and activator of transcription 3 and promoting adenosine monophosphate-activated protein kinase-autophagy for the balance between T helper 17 and Tregs. Transl. Res. 173, 115–130. 10.1016/j.trsl.2016.03.006 27126953

[B96] Paul W. E. (2015). History of interleukin-4. Cytokine 75, 3–7. 10.1016/j.cyto.2015.01.038 25814340 PMC4532601

[B97] Pelletier M. Maggi L. Micheletti A. Lazzeri E. Tamassia N. Costantini C. (2010). Evidence for a cross-talk between human neutrophils and Th17 cells. blood 115, 335–343. 10.1182/blood-2009-04-216085 19890092

[B98] Pérez-Jeldres T. Alvarez-Lobos M. Rivera-Nieves J. (2021). Targeting sphingosine-1-phosphate signaling in immune-mediated diseases: Beyond multiple sclerosis. Drugs 81, 985–1002. 10.1007/s40265-021-01528-8 33983615 PMC8116828

[B99] Proto J. D. Doran A. C. Gusarova G. Yurdagul A. Jr Sozen E. Subramanian M. Islam M. N. (2018). Regulatory T cells promote macrophage efferocytosis during inflammation resolution. Immunity 49, 666–677. 10.1016/j.immuni.2018.07.015 30291029 PMC6192849

[B100] Quinn P. J. (2014). Sphingolipid symmetry governs membrane lipid raft structure. Biochim. Biophys. Acta 1838, 1922–1930. 10.1016/j.bbamem.2014.02.021 24613791

[B101] Rådmark O. Werz O. Steinhilber D. Samuelsson B. (2015). 5-Lipoxygenase, a key enzyme for leukotriene biosynthesis in health and disease. Biochim. Biophys. Acta 1851, 331–339. 10.1016/j.bbalip.2014.08.012 25152163

[B102] Rajasagi N. K. Rouse B. T. (2018). Application of our understanding of pathogenesis of herpetic stromal keratitis for novel therapy. Microbes Infect. 20, 526–530. 10.1016/j.micinf.2017.12.014 29329934 PMC6037561

[B103] Regen S. L. (2020). The origin of lipid rafts. Biochemistry 59, 4617–4621. 10.1021/acs.biochem.0c00851 33226208

[B104] Romagnani S. (2014). T cell subpopulations. Chem. Immunol. Allergy 100, 155–164. 10.1159/000358622 24925396

[B105] Rudensky A. Y. (2011). Regulatory T cells and Foxp3. Immunol. Rev. 241, 260–268. 10.1111/j.1600-065X.2011.01018.x 21488902 PMC3077798

[B106] Sofi M. H. Heinrichs J. Dany M. Nguyen H. Dai M. Bastian D. (2017). Ceramide synthesis regulates T cell activity and GVHD development. JCI Insight 2. 10.1172/jci.insight.91701 PMC5436544 28515365

[B107] Sakaguchi S. Yamaguchi T. Nomura T. Ono M. (2008). Regulatory T cells and immune tolerance. Cell 133, 775–787. 10.1016/j.cell.2008.05.009 18510923

[B108] Salamone G. Giordano M. Trevani A. S. Gamberale R. Vermeulen M. Schettinni J. (2001). Promotion of neutrophil apoptosis by TNF-alpha. J. Immunol. 166, 3476–3483. 10.4049/jimmunol.166.5.3476 11207306

[B109] Salas A. Hernandez-Rocha C. Duijvestein M. Faubion W. McGovern D. Vermeire S. (2020). JAK-STAT pathway targeting for the treatment of inflammatory bowel disease. Nat. Rev. Gastroenterol. Hepatol. 17, 323–337. 10.1038/s41575-020-0273-0 32203403

[B110] Sallenave J-M. Guillot L. (2020). Innate immune signaling and proteolytic pathways in the resolution or exacerbation of SARS-CoV-2 in covid-19: Key therapeutic targets? Front. Immunol. 11, 1229. 10.3389/fimmu.2020.01229 32574272 PMC7270404

[B111] Sandborn W. J. Feagan B. G. D'Haens G. Wolf D. C. Jovanovic I. Hanauer S. B. (2021). Ozanimod as induction and maintenance therapy for ulcerative colitis. N. Engl. J. Med. 385, 1280–1291. 10.1056/NEJMoa2033617 34587385

[B112] Schauberger E. Peinhaupt M. Cazares T. Lindsley A. W. (2016). Lipid mediators of allergic disease: Pathways, treatments, and emerging therapeutic targets. Curr. Allergy Asthma Rep. 16, 48. 10.1007/s11882-016-0628-3 27333777 PMC5515624

[B113] Schnoor M. Alcaide P. Voisin M-B. van Buul J. D. (2016). Recruitment of immune cells into inflamed tissues: Consequences for endothelial barrier integrity and tissue functionality. Mediat. Inflamm. 2016, 1561368. 10.1155/2016/1561368 PMC4773555 26989330

[B114] Schwab S. R. Cyster J. G. (2007). Finding a way out: Lymphocyte egress from lymphoid organs. Nat. Immunol. 8, 1295–1301. 10.1038/ni1545 18026082

[B115] Selkirk J. V. Bortolato A. Yan Y. G. Ching N. Hargreaves R. (2022). Competitive binding of ozanimod and other sphingosine 1-phosphate receptor modulators at receptor subtypes 1 and 5. Front. Pharmacol. 13, 892097. 10.3389/fphar.2022.892097 35784713 PMC9247443

[B116] Sen P. Andrabi S. B. A. Buchacher T. Khan M. M. Kalim U. U. Lindeman T. M. (2021). Quantitative genome-scale metabolic modeling of human CD4+ T cell differentiation reveals subset-specific regulation of glycosphingolipid pathways. Cell Rep. 37, 109973. 10.1016/j.celrep.2021.109973 34758307

[B117] Shibuya M. (2011). Vascular endothelial growth factor (VEGF) and its receptor (vegfr) signaling in angiogenesis: A crucial target for anti- and pro-angiogenic therapies. Genes Cancer 2, 1097–1105. 10.1177/1947601911423031 22866201 PMC3411125

[B118] Shin S-H. Cho K-A. Yoon H-S. Kim S-Y. Kim H-Y. Pewzner-Jung Y. (2021). Ceramide synthase 2 null mice are protected from ovalbumin-induced asthma with higher T cell receptor signal strength in CD4+ T cells. Int. J. Mol. Sci. 22. 10.3390/ijms22052713 PMC7962461 33800208

[B119] Shiow L. R. Rosen D. B. Brdicková N. Xu Y. An J. Lanier L. L. (2006). CD69 acts downstream of interferon-alpha/beta to inhibit S1P1 and lymphocyte egress from lymphoid organs. Nature 440, 540–544. 10.1038/nature04606 16525420

[B120] Simons K. Sampaio J. L. (2011). Membrane organization and lipid rafts. Cold Spring Harb. Perspect. Biol. 3, a004697. 10.1101/cshperspect.a004697 21628426 PMC3179338

[B121] Smith N. C. Rise M. L. Christian S. L. (2019). A comparison of the innate and adaptive immune systems in cartilaginous fish, ray-finned fish, and lobe-finned fish. Front. Immunol. 10, 2292. 10.3389/fimmu.2019.02292 31649660 PMC6795676

[B122] Smith-Garvin J. E. Koretzky G. A. Jordan M. S. (2009). T cell activation. Annu. Rev. Immunol. 27, 591–619. 10.1146/annurev.immunol.021908.132706 19132916 PMC2740335

[B123] Sobrido-Cameán D. Barreiro-Iglesias A. (2018). Role of caspase-8 and Fas in cell death after spinal cord injury. Front. Mol. Neurosci. 11, 101. 10.3389/fnmol.2018.00101 29666570 PMC5891576

[B124] Solomou E. E. Keyvanfar K. Young N. S. (2006). T-bet, a Th1 transcription factor, is up-regulated in T cells from patients with aplastic anemia. blood 107, 3983–3991. 10.1182/blood-2005-10-4201 16434488 PMC1895294

[B125] Srivastava M. Baig M. S. (2018). NOS1 mediates AP1 nuclear translocation and inflammatory response. Biomed. Pharmacother. 102, 839–847. 10.1016/j.biopha.2018.03.069 29605772

[B126] Steinmetz O. M. Summers S. A. Gan P-Y. Semple T. Holdsworth S. R. Kitching A. R. (2011). The Th17-defining transcription factor RORγt promotes glomerulonephritis. J. Am. Soc. Nephrol. 22, 472–483. 10.1681/ASN.2010040435 21183590 PMC3060441

[B127] Stepanovska B. Huwiler A. (2020). Targeting the S1P receptor signaling pathways as a promising approach for treatment of autoimmune and inflammatory diseases. Pharmacol. Res. 154, 104170. 10.1016/j.phrs.2019.02.009 30776422

[B128] Sukocheva O. A. Lukina E. McGowan E. Bishayee A. (2020). Sphingolipids as mediators of inflammation and novel therapeutic target in inflammatory bowel disease. Adv. Protein Chem. Struct. Biol. 120, 123–158. 10.1016/bs.apcsb.2019.11.003 32085881

[B129] Szabo S. J. Kim S. T. Costa G. L. Zhang X. Fathman C. G. Glimcher L. H. (2000). A novel transcription factor, T-bet, directs Th1 lineage commitment. Cell 100, 655–669. 10.1016/s0092-8674(00)80702-3 10761931

[B130] Tabarkiewicz J. Pogoda K. Karczmarczyk A. Pozarowski P. Giannopoulos K. (2015). The role of IL-17 and Th17 lymphocytes in autoimmune diseases. Arch. Immunol. Ther. Exp. Warsz. 63, 435–449. 10.1007/s00005-015-0344-z 26062902 PMC4633446

[B131] Tao S. Tao R. Busch D. H. Widera M. Schaal H. Drexler I. (2019). Sequestration of late antigens within viral factories impairs MVA vector-induced protective memory CTL responses. Front. Immunol. 10, 2850. 10.3389/fimmu.2019.02850 31867011 PMC6904312

[B132] Thudichum J. L. W. (1962). A treatise on the chemical constitution of the brain.

[B133] Tikhonova A. N. van Laethem F. Hanada K-i. Lu J. Pobezinsky L. A. Hong C. (2012). αβ T cell receptors that do not undergo major histocompatibility complex-specific thymic selection possess antibody-like recognition specificities. Immunity 36, 79–91. 10.1016/j.immuni.2011.11.013 22209676 PMC3268851

[B134] Trapani J. A. (2001). Granzymes: A family of lymphocyte granule serine proteases. Genome Biol. 2, 3014.1–3014.7. 10.1186/gb-2001-2-12-reviews3014 PMC138995 11790262

[B135] Tsukuda Y. Iwasaki N. Seito N. Kanayama M. Fujitani N. Shinohara Y. (2012). Ganglioside GM3 has an essential role in the pathogenesis and progression of rheumatoid arthritis. PLoS One 7, e40136. 10.1371/journal.pone.0040136 22768242 PMC3387008

[B136] Turner J. E. Steinmetz O. M. Stahl R. A. Panzer U. (2007). Targeting of Th1-associated chemokine receptors CXCR3 and CCR5 as therapeutic strategy for inflammatory diseases. Mini Rev. Med. Chem. 7, 1089–1096. 10.2174/138955707782331768 18045212

[B137] Uche L. E. Gooris G. S. Bouwstra J. A. Beddoes C. M. (2021). Increased levels of short-chain ceramides modify the lipid organization and reduce the lipid barrier of skin model membranes. Langmuir 37, 9478–9489. 10.1021/acs.langmuir.1c01295 34319754 PMC8389989

[B138] Varela M. L. Mogildea M. Moreno I. Lopes A. (2018). Acute inflammation and metabolism. Inflammation 41, 1115–1127. 10.1007/s10753-018-0739-1 29404872

[B139] Vasandan A. B. Jahnavi S. Shashank C. Prasad P. Kumar A. Prasanna S. J. (2016). Human Mesenchymal stem cells program macrophage plasticity by altering their metabolic status via a PGE2-dependent mechanism. Sci. Rep. 6, 38308. 10.1038/srep38308 27910911 PMC5133610

[B140] Viallard J. F. Pellegrin J. L. Ranchin V. Schaeverbeke T. Dehais J. Longy-Boursier M. (1999). Th1 (IL-2, interferon-gamma (IFN-g)) and Th2 (IL-10, IL-4) cytokine production by peripheral blood mononuclear cells (PBMC) from patients with systemic lupus erythematosus (SLE). Clin. Exp. Immunol. 115, 189–195. 10.1046/j.1365-2249.1999.00766.x 9933441 PMC1905189

[B141] Voskoboinik I. Whisstock J. C. Trapani J. A. (2015). Perforin and granzymes: Function, dysfunction and human pathology. Nat. Rev. Immunol. 15, 388–400. 10.1038/nri3839 25998963

[B142] Walsh D. McCarthy J. O'Driscoll C. Melgar S. (2013). Pattern recognition receptors--molecular orchestrators of inflammation in inflammatory bowel disease. Cytokine Growth Factor Rev. 24, 91–104. 10.1016/j.cytogfr.2012.09.003 23102645

[B143] Wan Y. Y. (2010). Regulatory T cells: Immune suppression and beyond. Cell Mol. Immunol. 7, 204–210. 10.1038/cmi.2010.20 20383175 PMC2868372

[B144] Wang Y. Liu J. Burrows P. D. Wang J-Y. (2020). B cell development and maturation. Adv. Exp. Med. Biol. 1254, 1–22. 10.1007/978-981-15-3532-1_1 32323265

[B145] Watanabe S. Yamada Y. Murakami H. (2020). Expression of Th1/Th2 cell-related chemokine receptors on CD4+ lymphocytes under physiological conditions. Int. J. Lab. Hematol. 42, 68–76. 10.1111/ijlh.13141 31825162

[B146] Weirather J. Hofmann U. D. W. Beyersdorf N. Ramos G. C. Vogel B. Frey A. (2014). Foxp3+ CD4+ T cells improve healing after myocardial infarction by modulating monocyte/macrophage differentiation. Circ. Res. 115, 55–67. 10.1161/CIRCRESAHA.115.303895 24786398

[B147] Wiese T. Dennstädt F. Hollmann C. Stonawski S. Wurst C. Fink J. (2021). Inhibition of acid sphingomyelinase increases regulatory T cells in humans. Brain Commun. 3, fcab020. 10.1093/braincomms/fcab020 33898989 PMC8054263

[B148] Xie S. Z. Garcia-Prat L. Voisin V. Ferrari R. Gan O. I. Wagenblast E. (2019). Sphingolipid modulation activates proteostasis programs to govern human hematopoietic stem cell self-renewal. Cell Stem Cell 25, 639–653. e7. 10.1016/j.stem.2019.09.008 31631013 PMC6838675

[B149] Yamada A. Arakaki R. Saito M. Kudo Y. Ishimaru N. (2017). Dual role of fas/FasL-mediated signal in peripheral immune tolerance. Front. Immunol. 8, 403. 10.3389/fimmu.2017.00403 28424702 PMC5380675

[B150] Yamanaka K. Takahashi Y. Azuma Y. Hantani Y. (2020). Assay development and screening for the identification of ganglioside GM3 synthase inhibitors. Biochemistry 59, 1242–1251. 10.1021/acs.biochem.0c00055 32163271

[B151] Yanai H. Chiba S. Hangai S. Kometani K. Inoue A. Kimura Y. (2018). Revisiting the role of IRF3 in inflammation and immunity by conditional and specifically targeted gene ablation in mice. Proc. Natl. Acad. Sci. U. S. A. 115, 5253–5258. 10.1073/pnas.1803936115 29712834 PMC5960330

[B152] Yang S. Fujikado N. Kolodin D. Benoist C. Mathis D. (2015). Immune toleranceRegulatory T cells generated early in life play a distinct role in maintaining self-tolerance. Science 348, 589–594. 10.1126/science.aaa7017 25791085 PMC4710357

[B153] Yatim K. M. Lakkis F. G. (2015). A brief journey through the immune system. Clin. J. Am. Soc. Nephrol. 10, 1274–1281. 10.2215/CJN.10031014 25845377 PMC4491295

[B154] Ye J. (2013). Roles of regulated intramembrane proteolysis in virus infection and antiviral immunity. Biochim. Biophys. Acta 1828, 2926–2932. 10.1016/j.bbamem.2013.05.005 24099010 PMC3837687

[B155] Yoshie O. Matsushima K. (2015). CCR4 and its ligands: From bench to bedside. Int. Immunol. 27, 11–20. 10.1093/intimm/dxu079 25087232

[B156] Zhang L. Liu M. Liu W. Hu C. Li H. Deng J. (2021). Th17/IL-17 induces endothelial cell senescence via activation of NF-κB/p53/Rb signaling pathway. Lab. Invest. 101, 1418–1426. 10.1038/s41374-021-00629-y 34172831

[B157] Zhao X. Zhao Y. Sun X. Xing Y. Wang X. Yang Q. (2020). Immunomodulation of MSCs and MSC-derived extracellular vesicles in osteoarthritis. Front. Bioeng. Biotechnol. 8, 575057. 10.3389/fbioe.2020.575057 33251195 PMC7673418

[B158] Zhu Y. Gumlaw N. Karman J. Zhao H. Zhang J. Jiang J-L. (2011). Lowering glycosphingolipid levels in CD4+ T cells attenuates T cell receptor signaling, cytokine production, and differentiation to the Th17 lineage. J. Biol. Chem. 286, 14787–14794. 10.1074/jbc.M111.218610 21402703 PMC3083190

[B159] Zindel J. Kubes P. (2020). DAMPs, PAMPs, and LAMPs in immunity and sterile inflammation. Annu. Rev. Pathol. 15, 493–518. 10.1146/annurev-pathmechdis-012419-032847 31675482

[B160] Zingoni A. Soto H. Hedrick J. A. Stoppacciaro A. Storlazzi C. T. Sinigaglia F. (1998). Preferentially expressed in Th2 but not Th1 cutting edge: The chemokine receptor CCR8 is cells. J. Immunol. 161, 547–551. 9670926

[B161] Zumerle S. Molon B. Viola A. (2017). Membrane rafts in T cell activation: A spotlight on CD28 costimulation. Front. Immunol. 8, 1467. 10.3389/fimmu.2017.01467 29163534 PMC5675840

[B162] Zuo W. Chen Y-G. (2009). Specific activation of mitogen-activated protein kinase by transforming growth Factor-␤ receptors in lipid rafts is required for epithelial cell plasticity. Mol. Biol. Cell 20, 1020–1029. 10.1091/mbc.e08-09-0898 19056678 PMC2633387

